# Causal Role for Neutrophil Elastase in Thoracic Aortic Dissection in Mice

**DOI:** 10.1161/ATVBAHA.123.319281

**Published:** 2023-08-17

**Authors:** Mei Yang, Xinmiao Zhou, Stuart W.A. Pearce, Zhisheng Yang, Qishan Chen, Kaiyuan Niu, Chenxin Liu, Jun Luo, Dan Li, Yue Shao, Cheng Zhang, Dan Chen, Qingchen Wu, Pedro R. Cutillas, Lin Zhao, Qingzhong Xiao, Li Zhang

**Affiliations:** 1 Department of Cardiology, Institute for Developmental and Regenerative Cardiovascular Medicine, Xinhua Hospital Affiliated to Shanghai Jiaotong University School of Medicine, China (M.Y., Q.C., D.L., L. Zhang).; 2 Faculty of Medicine and Dentistry, William Harvey Research Institute (M.Y., X.Z., S.W.A.P., Z.Y., K.N., C.L., Q.X.), Queen Mary University of London, United Kingdom.; 3 Faculty of Medicine and Dentistry, Centre for Haemato-Oncology, Barts Cancer Institute (P.R.C.), Queen Mary University of London, United Kingdom.; 4 Department of Respiratory and Critical Care Medicine, Sir Run Run Shaw Hospital, Zhejiang University School of Medicine, Hangzhou, China (X.Z.).; 5 Department of Cardiothoracic Surgery, The First Affiliated Hospital of Chongqing Medical University, China (J.L., Y.S., C.Z., D.C., Q.W.).; 6 Department of Cardiology, Beijing Anzhen Hospital, Capital Medical University, China (D.L., L. Zhao).; 7 Key Laboratory of Cardiovascular Diseases, School of Basic Medical Sciences, Guangzhou Institute of Cardiovascular Disease, The Second Affiliated Hospital, Guangzhou Medical University, China (Q.X.).

**Keywords:** dissection, thoracic aorta, extracellular traps, inflammation, leukocyte elastase, phenotype

## Abstract

**BACKGROUND:**

Thoracic aortic dissection (TAD) is a life-threatening aortic disease without effective medical treatment. Increasing evidence has suggested a role for NE (neutrophil elastase) in vascular diseases. In this study, we aimed at investigating a causal role for NE in TAD and exploring the molecular mechanisms involved.

**METHODS:**

β-aminopropionitrile monofumarate was administrated in mice to induce TAD. NE deficiency mice, pharmacological inhibitor GW311616A, and adeno-associated virus-2–mediated in vivo gene transfer were applied to explore a causal role for NE and associated target gene in TAD formation. Multiple functional assays and biochemical analyses were conducted to unravel the underlying cellular and molecular mechanisms of NE in TAD.

**RESULTS:**

NE aortic gene expression and plasma activity was significantly increased during β-aminopropionitrile monofumarate–induced TAD and in patients with acute TAD. NE deficiency prevents β-aminopropionitrile monofumarate–induced TAD onset/development, and GW311616A administration ameliorated TAD formation/progression. Decreased levels of neutrophil extracellular traps, inflammatory cells, and MMP (matrix metalloproteinase)-2/9 were observed in NE-deficient mice. TBL1x (F-box-like/WD repeat-containing protein TBL1x) has been identified as a novel substrate and functional downstream target of NE in TAD. Loss-of-function studies revealed that NE mediated inflammatory cell transendothelial migration by modulating TBL1x-LTA4H (leukotriene A4 hydrolase) signaling and that NE regulated smooth muscle cell phenotype modulation under TAD pathological condition by regulating TBL1x-MECP2 (methyl CpG-binding protein 2) signal axis. Further mechanistic studies showed that TBL1x inhibition decreased the binding of TBL1x and HDAC3 (histone deacetylase 3) to *MECP2* and *LTA4H* gene promoters, respectively. Finally, adeno-associated virus-2–mediated *Tbl1x* gene knockdown in aortic smooth muscle cells confirmed a regulatory role for TBL1x in NE-mediated TAD formation.

**CONCLUSIONS:**

We unravel a critical role of NE and its target TBL1x in regulating inflammatory cell migration and smooth muscle cell phenotype modulation in the context of TAD. Our findings suggest that the NE-TBL1x signal axis represents a valuable therapeutic for treating high-risk TAD patients.

**Highlights**

- NE (neutrophil elastase) deficiency reduces thoracic aortic dissection (TAD) and increases animal survival.
- Pharmacological inhibition of NE prevents TAD onset and progression.
- NE promotes TAD development through proteolytic cleavage of F-box-like/WD repeat-containing protein TBL1x.
- NE regulates smooth muscle cell phenotype modulation under TAD pathological condition by regulating the TBL1x-MECP2 (methyl CpG-binding protein 2) signal axis.
- NE mediates inflammatory cell transendothelial migration by modulating TBL1x-LTA4H (leukotriene A4 hydrolase) signaling.

Thoracic aortic dissection (TAD)—a lethal aortic pathology with a high mortality and morbidity—is characterized by aortic intimal tearing with an incidence of 35/100 000 for those who are >65 years of age.^1,2^ Despite aortic aneurysm (AA; another lethal aortic disease) and TAD share multiple histopathologic alterations such as vascular smooth muscle cell (SMC) disappearance due to apoptosis or necrosis, medial areas of mucoid degeneration, and ECM (extracellular matrix) breakdown,^3^ no preexisting aneurysm was observed in most TAD cases (>80%).^2,4^ Moreover, animal study shows that TAD precedes formation of aneurysms and atherosclerosis induced by Ang II (angiotensin II) in ApoE-deficient mice,^5^ indicating distinct mechanisms underlying these 2 lethal aortic pathologies. Although dysregulation of TGFβ (transforming growth factor beta) and Ang II signaling,^6^ hypoxia and increased oxidative stress, imbalance of MMPs (matrix metalloproteinases) and their endogenous inhibitors (tissue inhibitors of metalloproteinases), and inflammation have been suggested to contribute to the pathogenesis of TAD,^2,3,7^ the underlying cellular and molecular mechanisms of TAD remain elusive.

NE (neutrophil elastase) is a serine protease with a primarily reported function in intracellular and extracellular pathogen destruction.^8^ It possesses potent proteolytic activity against various ECM proteins, as well as a variety of nonmatrix proteins such as cytokines/chemokines, cell surface proteins/receptors, and other functional soluble proteins.^8^ NE has been implicated in a variety of destructive and inflammatory diseases^8^ including chronic and acute lung diseases^9^ and cardiovascular diseases.^10–13^ Specifically, NE mRNA and protein were both detectable within human atherosclerotic plaques,^14^ and increased plasma NE activity was observed during atherosclerosis development.^15^ We^16^ and others^17^ have confirmed a causal role for NE in atherogenesis and injury-induced neointimal SMC hyperplasia.^18^ However, little is known about the potential involvement of NE in TAD. In this study, NE and ApoE double knockout mice (ApoE^−/−^/NE^−/−^ mice, named as NE-KO mice) and their littermates (ApoE^−/−^/NE^+/+^ mice, known as WT mice; both are on C57BL6/J background) generated in our previous study^16^ were used to establish the causal role of NE in TAD onset and progression, while a pharmacological NE inhibitor GW311616A was applied to explore the therapeutic potential of NE inhibition in treating TAD. We have demonstrated that NE plays a critical role in TAD formation and progression, and TBL1x (F-box-like/WD repeat-containing protein TBL1x) is a functional substrate of NE in the context of TAD.

## MATERIALS AND METHODS

### Materials and Data Availability

The data that support the findings of this study can be found in our article and Supplemental Material or are available from the corresponding author upon reasonable request.

### Materials

Antibodies against α-SMA (alpha smooth muscle actin; rabbit IgG, ab5694; mouse IgG, ab7817), SM22 (smooth muscle 22 alpha; rabbit IgG, ab14106), NE (rabbit IgG, ab68672), CD68 (cluster of differentiation; rabbit IgG, ab125212), Iba1 (ionized calcium-binding adaptor molecule 1; mouse IgG, ab15690), histone H3 (citrulline 2+8+17; rabbit IgG, ab5103), HDAC3 (histone deacetylase 3; rabbit IgG, ab137704), LTA4H (leukotriene A4 hydrolase; rabbit IgG, ab133512), MECP2 (methyl CpG-binding protein 2; rabbit IgG, ab253197), MMP-9 (mouse IgG, ab58803; rabbit IgG, ab283575), and MMP-2 (rabbit IgG, ab37150) were purchased from Abcam, United Kingdom. Antibodies against SMA (smooth muscle actin; mouse IgG, A5228) and α-tubulin (mouse IgG, T6074) were from Merck, United Kingdom. Antibody against TBL1x (mouse IgG, 66955-1-Ig) was from Proteintech Europe, United Kingdom. Antibody against Ly6G (lymphocyte antigen 6 complex locus G6D; rat IgG, 127602) was from Biolegend, United Kingdom. Antibody against MPO (myeloperoxidase) was from R&D Systems (goat IgG, AF3667). All secondary antibodies and other materials were from Thermo Fisher Scientific, Inc, United Kingdom, unless specifically indicated.

### Animal Experiments, Anesthesia, and Euthanasia

All animal experiments were conducted according to the Animals (Scientific Procedures) Act of 1986 (United Kingdom). All animal procedures were approved by the Queen Mary University of London Ethics Review Board (project license number: PP5521236) and conform to the guidelines from Directive 2010/63/EU of the European Parliament on the protection of animals used for scientific purposes or the National Institutes of Health guidelines (Guide for the Care and Use of Laboratory Animals). Age- and sex-matched mice were randomly allocated into experimental groups. For the β-aminopropionitrile monofumarate (BAPN)–induced TAD model, 3-week-old mice on C57BL6/J background from both sexes were fed a normal diet and administered vehicle (water) or a freshly prepared BAPN (A3134; Merck) solution dissolved in their drinking water (0.25% wt/vol) for up to 4 weeks, as described previously.^19–23^ To determine the therapeutic potential of NE inhibition in TAD, WT mice were randomly administered either vehicle or a pharmacological NE inhibitor GW311616A (G8419; 2 mg/kg by oral gavage, twice a week; Merck) starting at 1 day before BAPN administration as reported previously.^16,18^ Adeno-associated virus-2 (AAV2)–mediated specific gene transfer in aortic SMCs as described previously^20^ was conducted in WT and NE-KO mice to explore a potential role for the specific NE downstream target in NE-mediated TAD formation and onset. At the end of the protocol, all mice were euthanized by placing them under deep anesthesia with 100% O_2_/5% isoflurane, followed by decapitation.

### Collection of Human Aorta Tissue Specimens and Sera

Human thoracic aortic tissue specimens and sera were obtained from consenting patients at the time of elective surgery through a protocol approved by the Institutional Review Board of the First Affiliated Hospital of Chongqing Medical University between October 2020 and May 2021 (approval number: 2018-022-2) as described previously.^19,24^ Briefly, dissected human thoracic aortic tissues were collected from acute TAD patients who were free from any known connective tissue disorders during surgical operations, while healthy human thoracic aortic tissues were collected from organ donors who were free of any known aortic diseases. Moreover, sera were collected from age- and sex-matched acute TAD patients and normal heathy subjects and kept at −80 °C for future use. All patients gave their written informed consent before inclusion in the study, and all experiments were conducted according to the principles expressed in the Declaration of Helsinki. The basic clinical characteristics of the study population were described in our previous study.^24^

### Aortic Tissue Collection and Histopathologic Analysis

At the end of each animal experimental protocol, animals were euthanized, and the vascular tree was carefully exposed and dissected by laparotomy. Aortas were carefully inspected to document any gross pathologies including intimal/medial tearing/dissection, intramural hematoma (blood clot or yellow precipitates in the aortic wall), apparent focal dilation, and periaortic adhesion. Thoracic aortic tissues were collected and processed, followed by histopathologic analysis as described in our previous studies.^16,25^ Briefly, the aorta was fixed with 10% formalin for 24 hours at room temperature before being embedded in paraffin for sectioning. As described by Qi et al,^26^ cross sections (5.0 µm) were collected at locations 0, 100, and 200 µm from the proximal end of the TADs, with 0 µm defined as the point where the first complete circle was obtained during sectioning. One or 2 sections from each location were subjected to HE staining and elastin van Gieson staining, respectively. Degradation of medial elastic lamina was analyzed by elastin van Gieson staining using Elastic Stain Kit (Verhoeff Van Gieson; ab150667; Abcam) as per manufacturer instructions. Images were taken and elastin breaks (fragmentation) per section were manually counted by 2 experienced investigators blinded to the treatments.

### Aortic Tissue Immunofluorescence Staining

The procedure used for aortic immunofluorescence staining was similar to that described in our previous studies.^27–30^ Briefly, paraffin aortic sections were deparaffined with xylene and rehydrated with ethanol, followed by antigen retrieval in a Universal HIER antigen retrieval reagent (ab208572; Abcam). After blocking, the sections were incubated with indicated primary antibodies or respective IgG controls diluted in blocking buffer in a cold room (4 °C) overnight. The tissue sections were then washed and subsequently incubated with an appropriate Alexa Fluor Plus second antibody (1:1000 dilution), followed by nuclei staining with DAPI (4,6-diamidino-2-phenylindole; 1 µg/mL). After mounting, the slides were examined using a laser scanning confocal microscope (Zeiss LSM 510 Mark 4) and Zen 2009 image software. The mean fluorescence intensity for target proteins and DAPI of the aortic wall from each section were measured with the Image J pro software by 2 experienced investigators blinded to the treatments and presented as the relative mean fluorescence intensity (target proteins over DAPI). Alternatively, cells stained positive for the target proteins and DAPI (total cells) were counted by 2 experienced investigators blinded to the treatments. Three sections were analyzed per vessel sample and averaged.

### Bone Marrow Cell Isolation

Bone marrow monocytes^16,25^ and neutrophils^31^ were isolated as described previously. Briefly, bone marrow cells were harvested from mouse femurs and tibias and resuspended in Red Blood Cell Lysis Buffer (11814389001; Sigma) to lyse the red blood cells. Mouse bone marrow monocytes were isolated from the unlysed cells using a Monocyte Isolation Kit (130-100-629; Miltenyi Biotec) as per manufacturer instructions, while mouse bone marrow neutrophils were isolated using Histopaque-based density gradient centrifugation (Histopaque 1077/1119). The isolated cells were resuspended in RPMI-1640 (Roswell Park Memorial Institute) supplemented with 1% penicillin/streptomycin and subjected to respective analysis as indicted in the figure legends.

### Cell Culture and Treatment

C166 cells—a widely used mouse endothelial cell line—were purchased from ATCC (American Type Culture Collection; CRL-2581) and maintained in DMEM supplemented with 10% fetal bovine serum and 1% penicillin/streptomycin as per manufacturer instructions. THP-1 cells—a widely used human monocyte cell line—were purchased from ATCC (TIB-202) and cultured in ATCC-formulated RPMI-1640 medium (30-2001) supplemented with 0.05 mM 2-mercaptoethanol, 10% fetal bovine serum, and 1% penicillin/streptomycin as per manufacturer instructions. Primary murine aortic SMCs were isolated from 8-week-old WT or NE-KO mice with both sexes and routinely maintained in DMEM supplemented with 10% fetal bovine serum as described in our previous studies.^18,32–35^ Aortic SMCs between passages 3 and 8 were used in our study as described previously.^36^ Aortic SMCs were serum starved for 24 to 48 hours (0.5% fetal bovine serum), followed by various treatments as indicated for up to 48 hours.

### Lentivirus Generation and Infection

Lentiviral particles for gene-specific small hairpin RNAs (shRNAs) were generated as described previously.^34,36,37^ Gene-specific shRNA lentiviral particles were produced using MISSION shRNA plasmids (TRCN0000109359 for mouse *TBL1x*, TRCN0000118650 for human TBL1x, TRCN0000050867 for human *LTA4H*, MISSION shRNA Bacterial Glycerol Stock; Sigma) according to the protocol provided. The shRNA nontarget control vector (SHC002) was used as a negative control (sh-NT [non-targeting shRNA]). Briefly, 293T cells were transfected with the abovementioned shRNA plasmids and the packaging plasmids, pCMV-dR8.2 and pCMV-VSV-G (both obtained from Addgene), using TurboFect Transfection Reagent (Thermo Fisher Scientific, Inc) according to the manufacturer’s instructions. The supernatant containing the lentivirus was harvested 48 hours later, filtered, aliquoted, and stored at −80 °C. shRNA lentiviral infection was conducted as described previously with some modifications.^25,34,36–38^ Briefly, THP-1 cells or vascular SMCs were cultured in their respective culture medium overnight. One transducing unit per cell of control (sh-NT) or gene-specific shRNA lentivirus were added with 10 μg/mL hexadimethrine bromide (H9268; Sigma). After incubation for 40 to 48 hours, infected cells were subjected to various functional analysis as indicated.

### AAV2-shRNA-EGFP Generation and In Vivo Transfer

AAV2 containing a control nontarget (AAV2-sh-NT, sh-NT) or *Tbl1x* gene-specific (AAV2-sh-Tbl1x, sh-Tbl1x) shRNAs was synthesized by Hanbio Biotechnology, with following sequences: Sh-NT: top strand: GATCCGTTCTCCGAACGTGTCACGTAATTCAAGAGATTACGTGACACGTTCGGAGAATTTTTTC; bottom strand: AATTGAAAAAATTCTCCGAACGTGTCACGTAATCTCTTGAATTACGTGACACGTTCGGAGAACG. Sh-Tbl1x: top strand: AATTCGTAGCCAGCACCTTAGGTCAACATAACTCGAGTTATGTTGACCTAAGGTGCTGGCTATTTTTTG; bottom strand: GATCCAAAAAATAGCCAGCACCTTAGGTCAACATAACTCGAGTTATGTTGACCTAAGGTGCTGGCTACG.

These oligonucleotides were cloned into pHBAAV-U6-MCS-CMV-EGFP shRNA vector, and the resultant vectors were verified by DNA sequencing analysis. To generate AAV2-shRNA virus, shRNA plasmids were cotransfected with packaging plasmids (pAAV-RC [plasmid carrying AAV-2 replication and capsid genes] and pHelper) into 293T cells using Lipofiter transfection reagents (HB-TRCF-1000; Hanbio Biotechnology) according to the manufacturer’s instructions. AAV viral particles were harvested 3 days later and purified using the ViraTrap AAV Purification Maxiprep Kit (all serotypes, V1469-01; Biomiga) by following the detailed instructions provided in the kit. AAV2 viral titrations were measured by real-time quantitative polymerase chain reaction (RT-qPCR) analysis of the genomic copies of woodchuck hepatitis virus posttranscriptional regulatory element or inverted terminal repeats. Before injection, AAV2 shRNA virus was adjusted to 5×10^11^ vector genomes/mL using sterile saline, and 100 μL virus was injected into each mouse via tail vein.

### Real-Time Quantitative Polymerase Chain Reaction

RT-qPCR was performed as described previously.^33–36^ Briefly, total RNA was extracted from murine aortas or cells using Trizol reagent (Sigma) according to the manufacturer’s instructions and subjected to DNase I (Sigma) digestion to remove potential DNA contamination. cDNA was reversely transcribed from total RNAs using an Improm-II RT kit (Promega, Madison, WI) with RNase inhibitor (Promega) and Random primers (Promega) and diluted to a working concentration of 5 ng/μL. FS UNIVERSAL SYBR GREEN MASTERROX was used in RT-qPCR. RT-qPCR was performed on the CFXConnect Real-Time PCR Detection System (BioRad, United Kingdom) or LightCycler 480 Instrument (Roche, United Kingdom) for 96- or 384-well plates, respectively, using SYBR Green RT-qPCR master mix (Merck). The cycle threshold values were obtained using CFX Manager Software or LightCycler 480 software and later analyzed using the 2^−ΔΔCT^ method to determine relative changes in gene expression across all samples. Relative mRNA expression level was defined as the ratio of target gene expression level to 18S expression level, with that of the control sample set as 1.0. Primers were designed using the Primer Express software (Applied Biosystems), and the sequence for each primer was listed in Table S1.

### Western Blot Analysis

Equal amount of protein was separated by SDS-PAGE with 4% to 20% Tris-glycine gel (Invitrogen, Carlsbad, CA) and subjected to standard Western blot analysis. The blots were subjected to densitometric analysis with the Image J software. Relative protein expression level was defined as the ratio of target protein expression level to α-tubulin expression level with that of the control sample set as 1.0.

### Plasma NE Levels and Activity Analysis

Plasma NE levels were measured using a Human Neutrophil Elastase ELISA Kit (ab270204; Abcam) according to the manufacturer’s instructions. NE activity was measured using the Neutrophil Elastase Activity Assay Kit (Fluorometric; ab204730; Abcam) as per manufacturer instructions.

### MMP-2/9 Activity Analysis

Total and active MMP-2 and MMP-9 levels were assessed using the FRET (fluorescence resonance energy transfer) peptide–based immunocapture assay as described previously^39^ with modifications. Briefly, 500 ng of recombinant rabbit monoclonal anti-MMP-2 or anti-MMP-9 (ab181286 and ab228402; Abcam) was added to each well of a 96-well plate precoated with protein G (catalog number: 15157; Thermo Fisher Scientific, Inc) and incubated at 4 °C overnight. After washing, 50 μg aortic protein samples containing both active and pro-MMP-2/9 were added to each well and allowed to incubate with the antibody to facilitate tight binding. To quantify total MMP-2/9 levels, equal amount of 2 mM APMA (amino phenyl mercuric acetate) was added to activate pro-MMP-2/9 in the aortic protein samples. The captured MMP-2/9 activity was measured using the MMP Activity Assay Kit (Fluorometric-Red; ab112147; Abcam) as per manufacturer’s instructions. Specifically, 50 µL assay buffer was added into each well and preincubated at 37 °C for 10 minutes. Fifty microliters MMP red substrate working solution was added into each well and incubated at room temperature for 30 minutes. The fluorescence intensity was measured at excitation/emission of 540/590 nm using a Tecan microplate reader (Tecan Trading AG, Switzerland). Relative fluorescence unit was calculated by subtracting blank fluorescence readings from all measurements (control and treatments).

### Plasma CG, Trypsin, and Plasmin Activity

Cathepsin G Activity Assay Kit (Colorimetric; ab126780; Abcam), Trypsin Activity Colorimetric Assay Kit (MAK290‐1KT; Sigma, United Kingdom), or Plasmin Activity Assay Kit (Fluorometric; MAK244‐1KT; Sigma, United Kingdom) were used to detect plasma CG (cathepsin G), trypsin, and plasmin activity as per manufacturer’s instructions.

### Transendothelial Migration Assay

Similar to our previous study,^25^ bone marrow monocytes/neutrophils isolated from WT and NE-KO mice or THP-1 human monocytes were subjected to transendothelial migration assay using a trans-well plate with polycarbonate membrane inserts (8-μm pore size; Greiner Bio-One, Inc, United Kingdom). Briefly, 1×10^5^ bone marrow neutrophils/monocytes or THP-1 cells in 200 μL serum-free RPMI-1640 medium were placed over the inner chamber of inserts pregrown with a monolayer of C166 cells in a 24-well tissue culture plate, and 500 μL serum-free RPMI-1640 medium supplemented with 1% BSA and MIP2 (macrophage inflammatory protein 2; 100 ng/mL, for neutrophils) or MCP-1 (monocyte chemoattractant protein-1; 100 ng/mL for monocytes) were added into the bottom chamber of the insert. Plates were incubated at 37 °C for 4 hours. The cells that had migrated through to the lower surface of the insert were scraped off and mixed with the cells that had migrated into the bottom well. After collection, migrated cells were stained with 100 μL of 0.4% crystal violet solution for 20 minutes. After centrifuged to remove staining solution, the stained cells were dissolved in 100 μL 10% acetic acid, and absorbance (OD_560_) was detected at 560 nm using a Tecan microplate reader (Tecan Trading AG, Switzerland).

### Aortic Proteomics Analysis

Proteomics studies and data analysis were conducted as described in our previous studies.^18,29,32,40^ Briefly, the loosely bound newly synthesized proteins in aorta were specifically extracted and enriched using a similar method reported by Didangelos et al^41,42^ from both WT and NE-KO mice that received an Ang II infusion for 2 weeks. The extracted aortic proteins were digested using trypsin, and protein-derived peptides were analyzed by the LTQ Orbitrap XL mass spectrometer (Thermo Fisher Scientific, Inc) coupled with a nanoAcquity liquid chromatography system (Waters) as detailed in our previous study.^40^ The fold changes of protein expressions between WT and NE-KO aortas were transformed using the log_2_ function, while the *P* values were −log10 transformed for volcano plot analysis.

### Chromatin Immunoprecipitation Assay

The chromatin immunoprecipitation assays were performed as described in our previous studies.^33,34,36^ Briefly, WT or NE-KO vascular SMCs infected with control (sh-NT) or TBL1x-sepcific (sh-TBL1x) shRNA lentivirus were subjected to serum starvation for 24 hours, followed by incubation with 10 nM Ang II and 25 μg/mL BAPN for additional 48 hours. In another set of experiments, THP-1 cells were infected with sh-NT or sh-TBL1x lentivirus for 48 hours, followed by incubation with control vehicle or 40 nM NE inhibitor (GW311616A) for additional 24 hours. Cells were treated with 1% (v/v) formaldehyde at room temperature for 10 minutes and then quenched with glycine at room temperature. After then, the cells were harvested, sonicated, and immunoprecipitated with 5 µg IgG control or antibody against targeted proteins (anti-TBL1x, mouse IgG, 66955-1-Ig; anti-HDAC3, rabbit IgG, ab137704). Immunoprecipitated DNA was extracted, purified, and then used to amplify target DNA sequences by RT-qPCR. Promoter DNA enrichment with specific antibody was calculated using percentage input method with that of the IgG control set as 1.0. The relative level of promoter DNA enrichment was defined as the ratio of promoter DNA enrichments in the samples with the indicated treatments to the control samples (vehicle/sh-NT or WT/sh-NT) with that of the control sample set as 1.0.

### Statistical Analysis

All experiments were repeated at least for 3×. All data points represent independent biological but not technical replicates. Data collection and evaluation of all experiments was performed blinded to the group identity. Results are presented as mean±SEM. Statistical analysis was performed using Graphpad Prism (v9.1; GraphPad Software). The Shapiro-Wilk normality test and F test were used for checking the normality and homogeneity of variance of the data sets. Accordingly, 2-tailed unpaired Student *t* test was used for comparisons between 2 groups, or 1/2-way ANOVA with a post hoc test of Tukey analysis was applied when >2 groups were compared if the data displayed a normal distribution. Conversely, a nonparametric Mann-Whitney *U* test was applied for comparing 2 groups if the data did not display normal distribution. Additionally, log-rank (Mantel-Cox) test and χ^2^ test were applied to compare the survival rates and abdominal aortic aneurysm/TAD incidence among different groups, respectively. α=0.05 was chosen as the significance level, and a value of *P*<0.05 was considered as statistically significant.

## RESULTS

### NE Deficiency Prevents BAPN-Induced TAD Formation/Development

NE-deficient (NE-KO) mice and their control littermates (WT) on C57BL6/J genetic background were generated in our previous study.^16^ No apparent global phenotype was observed in NE-KO mice throughout their whole life cycle, and NE-KO mice displayed similar aortic anatomic characteristics to their WT control littermates under physiological condition (Figure S1). A widely reported murine TAD model induced through administration of BAPN in drinking water (0.25%, wt/vol)^19–23^ was utilized in this study to study a potential causal role of NE in TAD formation and onset. Initially, survival rate, TAD incidence (defined as the mice that died from thoracic aortic rupture and mice identified with ≥1 aortic pathologies [full-thickness aortic intima tear, penetrating aortic ulcer, false lumen, and intramural hematoma, which was deemed when red blood cells or thrombi were found between elastic laminae]), elastic fiber breaks within the aortic wall, and aortic NE gene expression were monitored in both male and female WT mice to determine the degree of sexual dimorphism in BAPN-induced TAD formation (Figure S2). However, we did not see any obvious phenotypic differences (Figure S2), suggesting that sex is not a determinant in the context of BAPN-induced TAD formation; therefore, all data from both sexes were pooled for statistical analysis in the following studies. Additionally, a time course study showed that BAPN-induced TAD mainly occurred within the third and fourth weeks of BAPN administration, with a cumulative TAD incidence of 85% at the end of the 4-week study protocol (Figure S2B). TAD was occasionally observed within the second week of treatment (<10%), and intramural hematomas were also observed in some mice (Figure S3A through S3C). Importantly, BAPN treatment induced significantly higher levels of both aortic *NE* gene expression (Figure S3D) and plasma NE activity (Figure S3E), with a peak level at 2 weeks post-BAPN administration. These data further suggest an involvement of NE in TAD formation and development.

**Figure 1.**
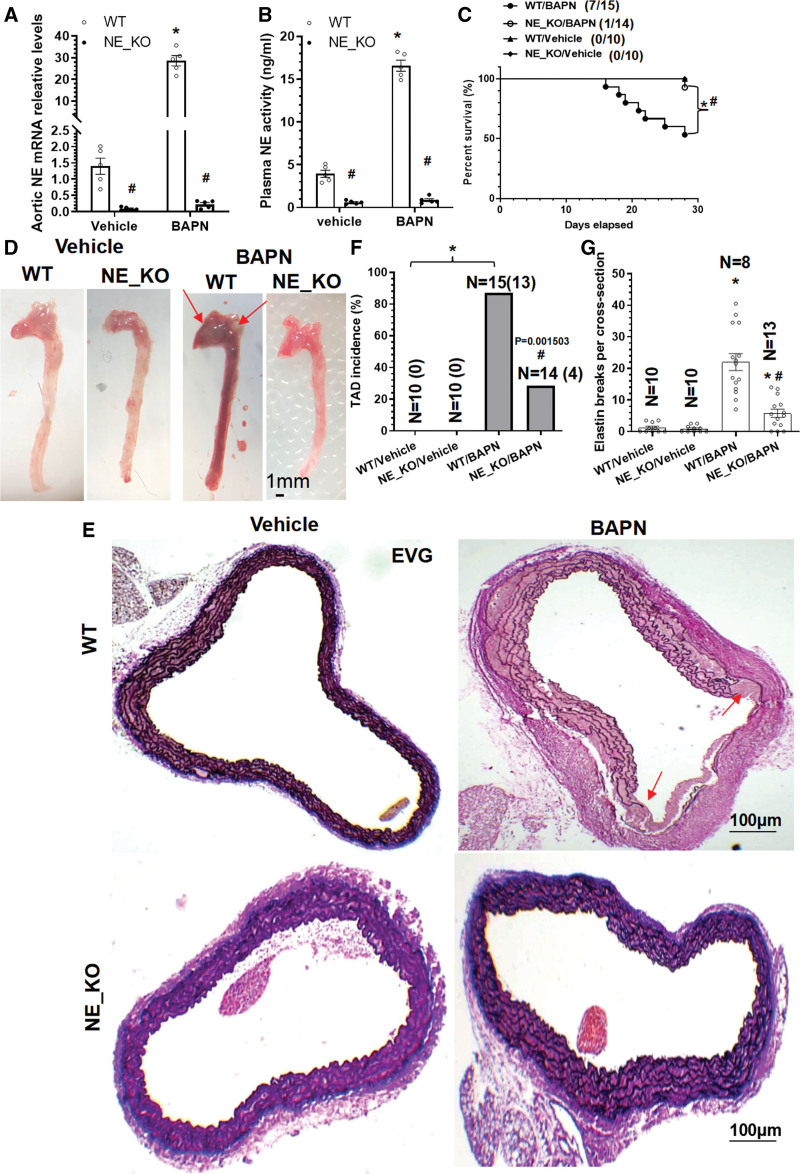
**NE (neutrophil elastase) plays a critical role in β-aminopropionitrile monofumarate (BAPN)–induced thoracic aortic dissection (TAD) formation.** Three-week-old *ApoE*^−/−^/*NE*^+/+^ (WT) or *ApoE*^−/−^/*NE*^−/−^ (NE-KO) mice from both sexes were randomly administered with vehicle (water) or 0.25% BAPN (wt/vol) in drinking water for 2 (**A** and **B**) or 4 (**C–G**) weeks, respectively. **A**, Thoracic aortic gene expression was analyzed by real-time quantitative polymerase chain reaction (RT-qPCR). **B**, Plasma NE activity was measured using a commercially available kit (ab204730). **C**, Kaplan-Meier survival curve. 7/15 indicates 7 of 15 mice were dead. **D**, Macroscopic images of aortas. Thoracic aortas were collected and subjected to elastin van Gieson (EVG) staining (**E**) analysis. The quantitative data of TAD incidence (**F**; n=10 (0) indicates that none of the 10 mice has TAD) and elastin breaks (**G**) were presented here. TAD incidence was defined by the mice died from aortic rupture, and mice identified with ≥1 aortic pathologies (aortic intima tear, false lumen, intramural hematoma). Red arrow indicates TAD site. Data presented here are representative (**D** and **E**) or mean±SEM of 5 (n=5 mice in **A** and **B**) or the indicated numbers (shown in **C**, **F**, and **G**) of mice, respectively. **A**, **B**, and **G**, *P<0.001 (versus vehicle), #*P*<0.001 (versus WT), 2-way ANOVA with a post hoc test of Tukey analysis. **C**, *P<0.001 (versus vehicle), #*P*<0.001 (versus WT), log-rank (Mantel-Cox) test. **F**, *P<0.001 (versus vehicle), #*P*=0.001503 (versus WT), χ^2^ test.

To confirm such a role, both WT and NE-KO mice were subjected to BAPN administration. Expectedly, while both NE aortic expression levels (Figure 1A) and plasma activities (Figure 1B) were absent or extremely low in NE-KO mice, BAPN administration in WT mice significantly upregulated their expression. Importantly, while no mortality was observed in both WT and NE-KO mice with vehicle treatment, 7 of 15 WT mice and 1 of 14 NE-KO mice treated with BAPN died due to aortic rupture during the 4-week treatment protocol (Figure 1C). Moreover, compared with WT mice, a lower TAD incidence and decreased levels of elastic fiber breaks were observed in NE-KO mice treated with BAPN (Figure 1D through 1G), confirming a causal role for NE in TAD onset and aortic rupture.

### TAD Formation and Aortic Rupture Is Prevented by NE Pharmacological Inhibition

**Figure 2.**
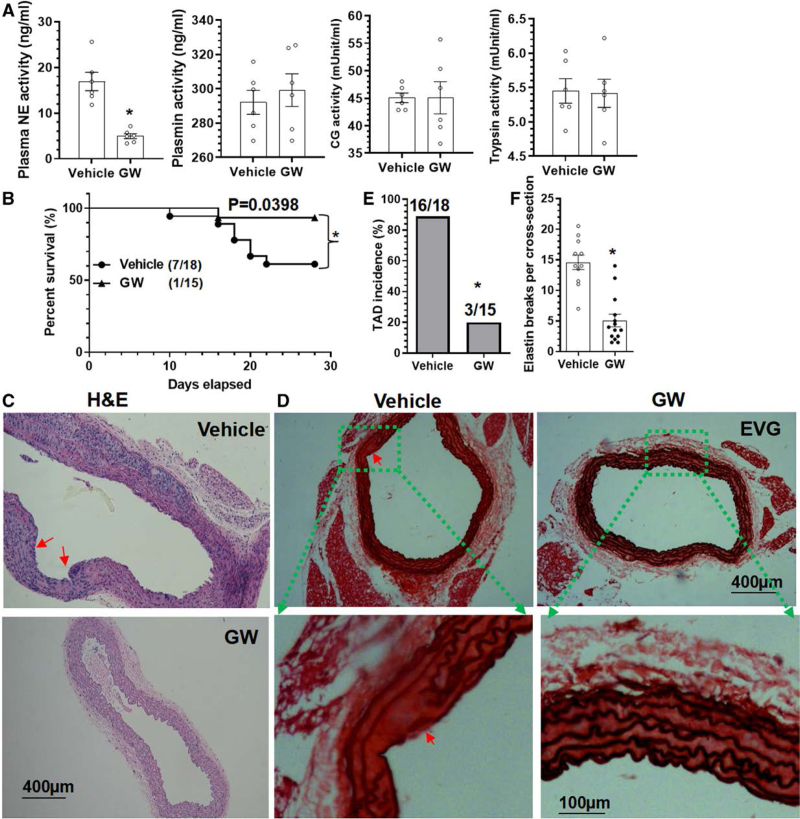
**Pharmacological inhibition of NE (neutrophil elastase) decreased β-aminopropionitrile monofumarate (BAPN)–induced AD formation.** Three-week-old *ApoE*^−/−^/*NE*^+/+^ (WT) mice from both sexes that received BAPN in drinking water were randomly administered with a vehicle control (vehicle) or NE inhibitor (GW311616A [GW]) for 2 (**A**) or 4 (**B–F**) weeks, respectively. **A**, Plasma NE, CG (cathepsin G), trypsin, and plasmin activity. **B**, Animal survival curves. 7/18 indicates 7 of 18 mice were dead. **C** through **F**, Representative images for HE staining (**C**), elastin van Gieson (EVG) staining (**D**), and the quantitative data of thoracic aortic dissection (TAD) incidence (**E**, 16/18 indicates 16 of 18 mice have TAD) and elastin breaks (**F**) were included here. Red arrows indicate TAD or intima tear. Data presented here are representative (**C** and **D**) or mean±SEM of 6 (**A**) or 11 (vehicle)/14 (GW) mice (**F**), respectively (n=6 or 11/14 mice). *P<0.001 (versus vehicle, unpaired *t* test in **A** and **F**), *P*=0.0398 (log-rank [Mantel-Cox] test in **B**), and *P<0.001 (versus vehicle, χ^2^ test in **E**).

Similar to our previous studies,^16,18^ the pharmacological NE inhibitor GW311616A was administrated into WT mice on a C57BL6/J genetic background to explore the therapeutic potential of NE inhibition in BAPN-induced TAD formation. We found that while GW311616A significantly inhibited plasma NE activity, GW311616A administration produced no significant effects on several other protease activities including CG, trypsin, and plasmin (Figure 2A). Functionally, GW311616A administration dramatically reduced mortality due to aortic rupture (Figure 2B) and decreased the TAD incidence and elastin fragmentation (Figure 2C through 2F). These data collectively demonstrate that pharmacological inhibition of NE with GW311616A inhibits TAD onset and prevents aortic rupture.

### NE Deficiency Reduces Formation of Neutrophil Extracellular Traps

NE has been reported to play a critical role in the formation of neutrophil extracellular traps (NETs),^43^ and we have previously found that NE-mediated NETs participate in injury-induced arterial remodeling.^18^ Accordingly, it is plausible for us to hypothesize that *NE* gene inactivation might also impair the formation of NETs during BAPN-induced TAD formation. Indeed, immunofluorescence staining of citrullinated histone H3 and MPO—2 surrogate biomarkers for NETs—showed that the formation of NETs in the dissected aorta was significantly reduced in NE-KO mice compared with WT mice (Figure S4A and S4B). A similar phenomenon was observed in neutrophils isolated from WT or NE-KO mice upon phorbol-1-myristate-13-acetate stimulation (Figure S4C and S4D). Taken together, the above evidence clearly supports a role for NE in the formation of NETs in the context of BAPN-induced TAD formation and progression.

### NE Gene Inactivation Reduces Aortic Inflammatory Cell Accumulation and Decreases Aortic MMP Expression and Activity

Immunofluorescence (IF) staining analyses with different antibodies were performed in aortic tissues from mice that had received 2 weeks of BAPN administration. We observed that abundant Ly6G^+^ neutrophils and Iba1^+^ macrophages were accumulated in the WT aorta, while much less inflammatory cells were observed in NE-KO aorta (Figure S5A through S5F). Moreover, IF staining data showed a significant decrease in both MMP-2 and MMP-9 protein expression in NE-KO aorta (Figure S5A through S5F). Importantly, we found significantly lower levels of both total and active aortic MMP-2, as well as MMP-9 in NE-KO mice, when compared with WT mice (Figure S5G). Similarly, we observed that compared with WT mice, the gene expression levels of both inflammatory genes (*MCP*-1, *IL* [interleukin]-*12*β**, and *IL-6*) and adhesion molecules (*ICAM1* [intercellular adhesion molecule 1] and *VCAM1* [vascular cell adhesion molecule 1]) in aorta were significantly reduced in NE-KO mice (Figure S5H).

### TBL1x Protein Is a Novel Substrate of NE

**Figure 3.**
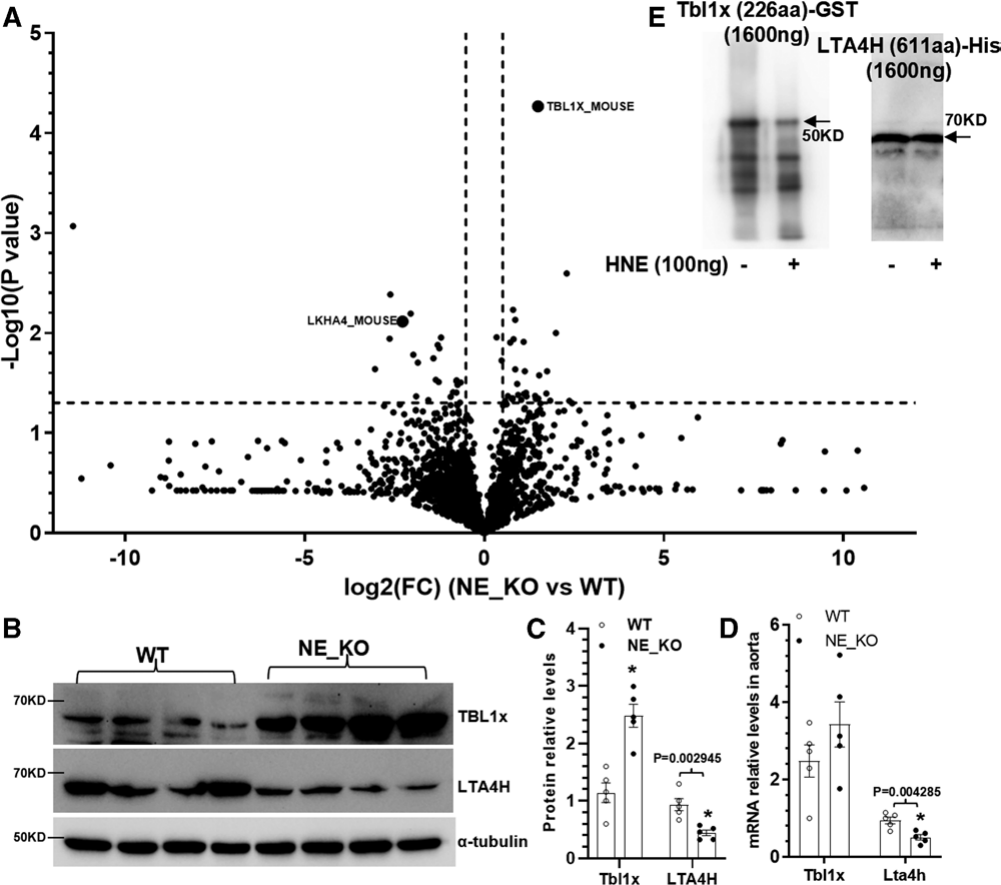
**Increased levels of TBL1x (F-box-like/WD repeat-containing protein TBL1x) and decreased expression levels of LTA4H (leukotriene A4 hydrolase) were observed in ApoE_−/−_/NE_−/−_ (NE-KO) aorta. A**, Volcano plot. Eight-week-old male *ApoE*^−/−^/*NE*^+/+^ (WT) or NE-KO mice were infused with saline or Ang II (angiotensin II; 1000 ng/kg per min) by osmotic minipump (model 2004; Durect) for 2 weeks. Abdominal aortic proteins were extracted and subjected to label-free quantitative proteomics analysis. Volcano plot showing the *P* values (−log10) against the fold changes of protein expression levels (log2) in NE-KO aortas vs WT aortas. **B** through **D**, Thoracic aortic proteins and RNAs were extracted from WT and NE-KO male mice treated with β-aminopropionitrile monofumarate (BAPN) for 2 weeks and subjected to Western blot (**B** and **C**) and real-time quantitative polymerase chain reaction (RT-qPCR; **D**) analysis, respectively. The data presented here are representatives (**B**) or mean±SEM (**C** and **D**) of 5 (n=5) mice. *P<0.001 (versus WT, unpaired *t* test); if any analysis with *P*<0.05, but *P*>0.001, the exact *P* value was included in respective figures. **E**, Tbl1x and LTA4H in vitro digestion. Recombinant human TBL1x and LTA4H proteins (1600 ng) were incubated with activated HNE (human neutrophil elastase; 100 ng) in assay buffer for 30 minutes, followed by the Western blot analysis with TBL1x and LTA4H antibody, respectively. Representative images from 3 independent experiments (n=3) were presented here.

NE was found to play a role in Ang II–induced abdominal AA (data not shown) in a parallel study, and abdominal aortic proteomics analysis was performed to identify possible target proteins of NE in such aortic pathology. As shown in Figure S6, 132 and 24 proteins were found to be significantly upregulated and downregulated, respectively, in NE-KO aorta in response to 2 weeks of Ang II infusion. A volcano plot (Figure 3A) showed that among the differentially expressed proteins, TBL1x (also known as TBL1) was the most significantly upregulated protein in NE-KO aorta in response to Ang II infusion. The upregulation of TBL1x protein in thoracic aortic tissues of NE-KO mice administrated with BAPN for 2 weeks was also confirmed by Western blot analysis (Figure 3B and 3C). Interestingly, we found no significant change in terms of thoracic aortic *TBL1x* gene expression between WT and NE-KO mice in response to BAPN administration (Figure 3D), suggesting a posttranscriptional regulation of TBL1x by NE under TAD pathological conditions. On the contrary, the LTA4H (or LKHA4 [leukotriene A4 hydrolase]) protein level was significantly downregulated in NE-KO aorta under BAPN treatment (Figure 3A through 3C). Unlike *TBL1x*, *LTA4H* gene expression was also significantly decreased in NE-KO aorta (Figure 3D). Importantly, data from in vitro protein digestion assay showed that NE could directly cleave TBL1x but not LTA4H (Figure 3E). These data collectively indicate TBL1x is one of the novel NE substrates in the context of TAD.

### NE Mediates Inflammatory Cell Transendothelial Migration by Regulating the Tbl1x/Lta4h Signal Axis

**Figure 4.**
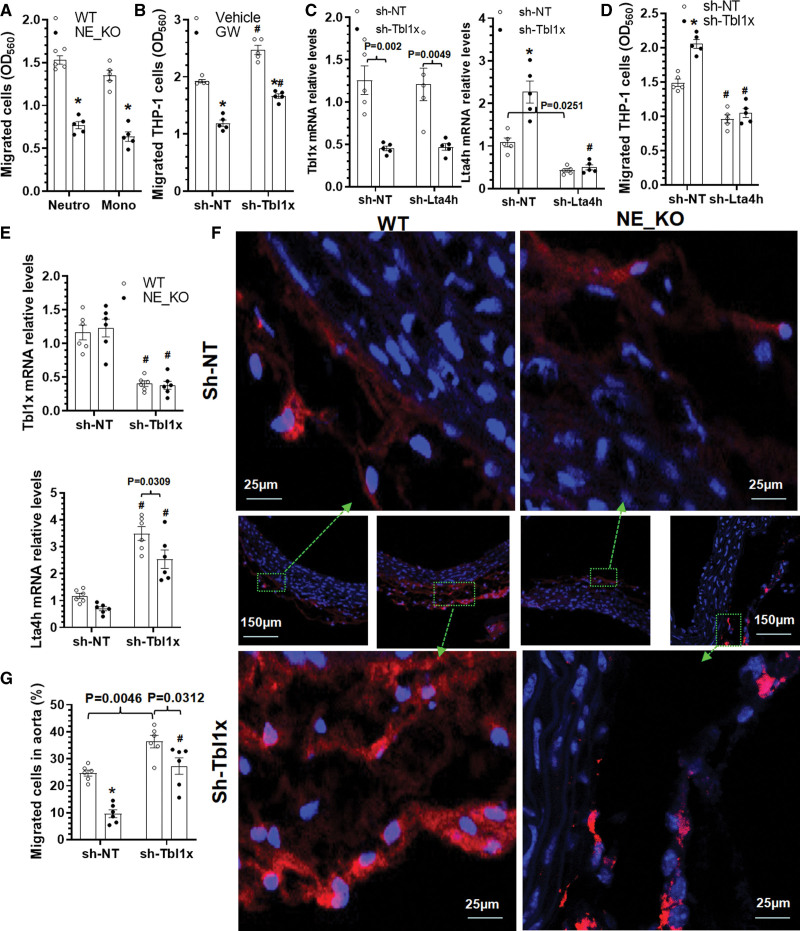
**NE (neutrophil elastase) mediates inflammatory cell transendothelial migration by regulating TBL1x (F-box-like/WD repeat-containing protein TBL1x)/LTA4H (leukotriene A4 hydrolase) signal axis. A**, Bone marrow neutrophils or monocytes were isolated from *ApoE*^−/−^/*NE*^+/+^ (WT) or *ApoE*^−/−^/*NE*^−/−^ (NE-KO) mice and subjected to transendothelial migration assay. Mouse endothelial cells (ECs; C166 cells) were precultured onto trans-well inserts (pore size, 8 µm) to form an EC monolayer, followed by transendothelial assays in response to 100 ng/mL MIP2 (macrophage inflammatory protein 2) for neutrophils or MCP-1 (monocyte chemoattractant protein-1) for monocytes, respectively. **B**, THP-1 cell transendothelial migration analysis in response to MCP-1. THP-1 cells infected with control (sh-NT [non-targeting shRNA]) or Tbl1x gene-specific (sh-Tbl1x) small hairpin RNA (shRNA) lentivirus were subjected to transendothelium migration assay in the absence or presence of NE inhibitor (GW311616A [GW], 40 nM). **C**, Real-time quantitative polymerase chain reaction (RT-qPCR) analysis showing *Tbl1x* and *Lta4h* gene expressions in THP-1 cells. **D**, TBL1x mediated THP-1 cell transendothelial migration through regulation of LTA4H. THP-1 cells infected with control (sh-NT) or gene-specific (sh-Tbl1x or sh-Lta4h) shRNA lentivirus were subjected to transendothelium migration assay. **E** through **G**, NE mediates monocyte recruitment into aortic walls by regulating the TBL1x/LTA4H signal axis. Bone marrow monocytes isolated from age- and sex-matched WT or NE-KO mice primed with β-aminopropionitrile monofumarate (BAPN) for 2 weeks were infected with control (sh-NT) or Tbl1x gene-specific (sh-Tbl1x) shRNA lentivirus. While a proportion of monocytes was subjected to RT-qPCR analysis (**E**), the rest of cells were labeled with cell tracker orange (C2927; Invitrogen) and randomly injected into age- and sex-matched NE-KO mice preadministrated with BAPN for 2 weeks via tail vein (5×10^6^ cells per mouse). Twenty-four later, aortas were collect for analysis (**F** and **G**). Data presented here are representatives (**F**) or mean±SEM of 5 (n=5; **A–D**) or 6 (n=6; **E** and **G**) independent experiments/mice. *P<0.001 (versus WT, vehicle, or sh-NT) and #*P*<0.001 (versus sh-NT); if any analysis with *P*<0.05, but *P*>0.001, the exact *P* value was included in respective figures. **A**, Unpaired *t* test. **B** through **D**, **E**, and **G**, Two-way ANOVA with a post hoc test of Tukey analysis.

We have previously shown that NE gene inactivation reduces aortic inflammatory cell accumulation during BAPN-induced TAD formation (Figure S5A through S5F). Moreover, IF staining analysis with antibodies for SMA, Ly6G, and NE in the aortic tissues obtained from WT mice administrated with BAPN for 3 weeks showed that there were a large number of Ly6G^+^ neutrophils expressing NE within the aortic media layer and at dissection sites (Figure S7), inferring a role for NE in inflammatory cell migration and recruitment into the aortic wall upon BAPN treatment. Indeed, data from transendothelial migration assays showed that NE-deficient neutrophils and monocytes displayed a decreased transendothelial migratory capacity in response to MIP2 or MCP-1 stimulations, respectively (Figure 4A), confirming an important role for NE in inflammatory cell migration and recruitment into the aortic wall under TAD pathological conditions. Moreover, IF staining data showed that TBL1x was highly expressed in CD68^+^ cells in the aorta (Figure S8). Importantly, we found that *TBL1x* knockdown significantly increased THP-1 monocyte transendothelial migration, but this promotive effect was lost when NE activity was inhibited by GW311616A (Figure 4B), indicating a functional role for TBL1x in NE-mediated monocyte migration.

Interestingly, since the principal function of LTA4H is converting leukotriene A4 to leukotriene B4, which is a potent chemoattractant for inflammatory cells in inflammatory diseases,^44,45^ we asked whether TBL1x mediated inflammatory cell migration through regulation of LTA4H. To answer this question, THP-1 monocytes were coinfected with *TBL1x* and *LTA4H* gene-specific shRNA lentivirus. As expected, *TBL1x* and *LTA4h* gene expressions were specifically inhibited by their respective shRNAs (Figure 4C). However, we observed that while TBL1x gene knockdown significantly upregulated *LTA4H* gene expression, no reciprocal regulatory effect of LTA4H knockdown on *TBL1x* gene expression was observed (Figure 4C), confirming that *LTA4H* is a downstream regulatory gene of TBL1x. Functional studies showed that while the transendothelial migratory ability of THP-1 monocytes in response to MCP-1 stimulation was significantly increased and decreased by knockdown of *TBL1x* and *LTA4H*, respectively, the promotive effect of *TBL1x* knockdown on monocyte migration was blunted by *LTA4H* inhibition (Figure 4D), suggesting a critical role for TBL1x/LTA4H signaling in NE-mediated inflammatory cell transendothelial migration.

To further confirm an in vivo role for TBL1x in NE-mediated inflammatory cell recruitment into aortic wall under TAD pathology, WT and NE-deficient bone marrow monocytes isolated from BAPN-treated mice were infected with control and *TBL1x* gene-specific shRNA lentivirus. After culturing for 24 hours, bone marrow monocytes were transplanted into NE-KO mice pretreated with BAPN for 2 weeks through tail vein injection. Gene expression data showed decreased levels of *LTA4H* in NE-KO bone marrow monocytes, while *Tbl1x* gene knockdown significantly increased *LTA4H* expression levels in both WT and NE-KO bone marrow monocytes (Figure 4E). Consequently, we observed that while *NE* gene deficiency and *Tbl1x* gene silencing could significantly decrease and increase bone marrow monocyte recruitment into aortic wall, respectively, the inhibitory effect of *NE* gene inactivation on bone marrow monocyte recruitment disappeared when the *Tbl1x* gene was silenced (Figure 4F and 4G). It is important to note that accumulation of adoptively transferred bone marrow monocytes in aorta is likely impacted by multiple factors, such as the rate of survival and off-target homing to other tissues including the lung, liver, and spleen. Nonetheless, the above data suggest that the TBL1x-LTA4H signal axis is at least partially responsible for NE-mediated inflammatory cell recruitment into aortic wall in the context of TAD pathology.

### NE Mediates SMC Phenotypic Modulation Under TAD Pathological Conditions by Regulating the TBL1x/MECP2 Signal Axis

**Figure 5.**
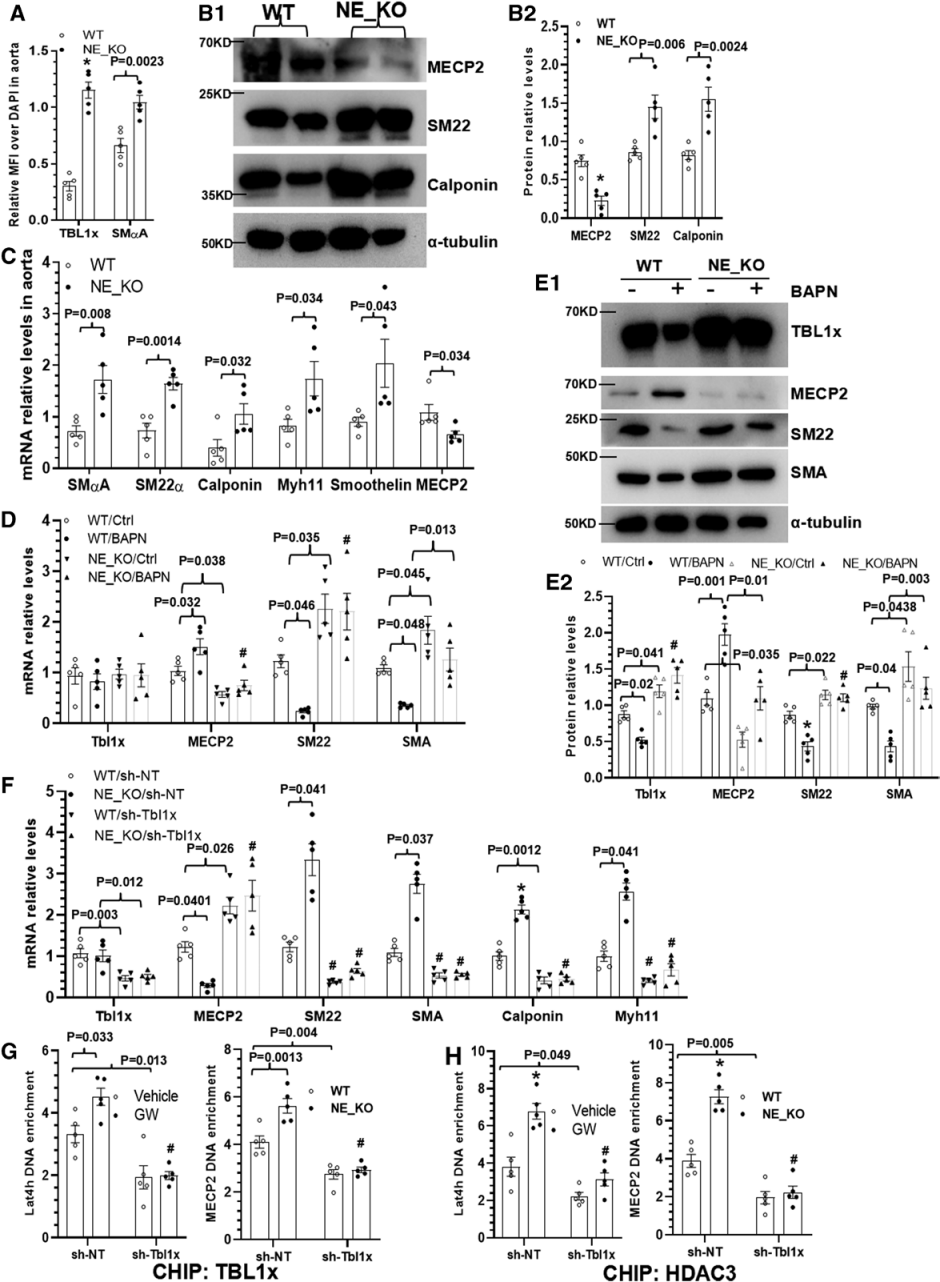
**NE (neutrophil elastase) mediates smooth muscle cell (SMC) phenotypic modulation under thoracic aortic dissection (TAD) pathological condition by regulating the TBL1x (F-box-like/WD repeat-containing protein TBL1x)/MECP2 (methyl CpG-binding protein 2) signal axis. A**, Quantitative analysis of immunofluorescence (IF) staining showing increased TBL1x protein expression in thoracic aortas. **B** and **C**, Three-week-old *ApoE*^−/−^/*NE*^+/+^ (WT) and *ApoE*^−/−^/*NE*^−/−^ (NE-KO) mice were administered with β-aminopropionitrile monofumarate (BAPN) in drinking water (0.25% wt/vol) for 2 weeks, thoracic aortic proteins and RNAs were exacted and subjected to Western blot (**B**) and real-time quantitative polymerase chain reaction (RT-qPCR; **C**) analysis, respectively. **D** and **E**, NE deficiency prevents Ang II (angiotensin II)/BAPN-induced SMC phenotypic switching. Serum-starved WT or NE-KO SMCs were treated with 10 nM Ang II and control (Ctrl) or 25 μg/mL BAPN for 48 hours. Total RNAs and cell lysates were collected and subjected to RT-qPCR (**D**) and Western blot (**E**) analysis, respectively. **F**, TBL1x is required for NE-mediated SMC gene regulation. WT or NE-KO SMCs infected with control (sh-NT [non-targeting shRNA]) or Tbl1x (sh-Tbl1x) small hairpin RNAs (shRNAs) were treated with 10 nM Ang II and 25 μg/mL BAPN for 48 hours. Total RNAs were collected and subjected to RT-qPCR analysis. **G** and **H**, Chromatin immunoprecipitation (CHIP) assays showing TBL1x inhibition decreases the direct binding of TBL1x (**G**) and HDAC3 (histone deacetylase 3; **H**) to *MECP2* and *LTA4H* (leukotriene A4 hydrolase) gene promoters, respectively. The data presented here are representatives (B1 and E1) or mean±SEM of 5 mice (**A**) or 5 independent experiments (**B–H**, n=5). *P<0.001 (versus WT, WT/Ctrl, or WT/sh-NT) and #*P*<0.001 (versus WT/BAPN, NE-KO/sh-NT, or GW311616A [GW]/sh-NT); if any analysis with *P*<0.05, but *P*>0.001, the exact *P* value was included in respective figures. **A** through **C**, Unpaired *t* test. **D** through **H**, Two-way ANOVA with a post hoc test of Tukey analysis.

Since SMC phenotypic switching or modulation has been identified as one of the fundamental mechanisms underlying AA and dissection formation and progression,^46–48^ we wondered whether a similar mechanism was underpinning NE-mediated AA and dissection formation. To test such a mechanism, we first examined NE protein expression in aortic cells and found that NE was highly expressed in aortic SMCs under TAD pathological conditions (Figure S9A). Moreover, we observed Ang II increased *NE* gene expression in cultured aortic SMCs, which was further significantly upregulated by addition of BAPN in a dose-dependent manner (Figure S9B). Similar regulatory effects of Ang II and BAPN on NE activity in aortic SMC cell lysate (Figure S9C) and conditioned culture medium (Figure S9D) were observed. As described previously (Figure S9A), IF staining data showed that TBL1x protein was also highly expressed in aortic SMCs during BAPN-induced TAD formation, with a higher expression level in NE-KO mice (Figure 5A; Figure S10). Interestingly, a higher expression level of SMC-specific proteins (SM22 and calponin) was observed in NE-KO aortas (Figure 5B). Gene expression data also showed that compared with WT controls, the expression levels of multiple SMC-specific genes were significantly increased in NE-KO aortas in response to BAPN treatment (Figure 5C), but no such difference was observed in NE-KO aorta treated with vehicle control (Figure S11A), inferring a regulatory role for NE in SMC phenotypic switching under TAD pathological conditions.

Since we have previously reported that increased Ang II production underpins the promotive effect of MMP-8 on BAPN-induced TAD formation,^19^ we wondered whether we could observe a similar phenomenon in NE-mediated TAD formation. While BAPN administration significantly increased plasma Ang II levels, no difference was observed between WT and NE-KO mice (Figure S11B). Moreover, since we observed a synergistic effect of Ang II and BAPN on NE expression and activity in SMCs (Figure S9B through S9D), we examined whether a similar synergistic effect of Ang II and BAPN occurred on SMC gene expression. Indeed, data showed that BAPN alone had no obvious effect on multiple SMC-specific gene expressions (Figure S11C), while a dramatic inhibitory effect was documented in the presence of 10 nM Ang II (Figure S11D), showing a synergistic effect of Ang II and BAPN on SMC gene regulation. Accordingly, WT and NE-KO SMCs were incubated with Ang II in the absence or presence of BAPN to further confirm the regulatory role of NE in SMC phenotypic modulation. We observed that compared with Ang II treatment alone, Ang II/BAPN combinational treatment significantly decreased SMC gene expressions in WT but not in NE-KO SMCs (Figure 5D), which was further confirmed at the protein level (Figure 5E). Importantly, TBL1x knockdown dramatically downregulated SMC gene expressions in both WT and NE-KO aortic SMCs (Figure 5F), illustrating a critical regulatory role of TBL1x in NE-mediated SMC phenotypic modulation under TAD pathological conditions.

Since MECP2 has been previously identified as a key suppressor of SMC gene expression in both stem cell–derived SMCs^49^ and aortic SMCs,^36^ we, therefore, speculated that MECP2 might play a role in NE-mediated SMC phenotypic switching under TAD pathological conditions. Indeed, we observed that both MECP2 gene (Figure 5C) and protein (Figure 5B) expression levels in the aorta were significantly decreased in NE-KO mice treated with BAPN for 2 weeks. Moreover, Ang II/BAPN cotreatment significantly increased MECP2 expression in WT but not in NE-KO aortic SMCs at both the gene and protein levels (Figure 5D and 5E). Interestingly, *MECP2* gene expression was significantly upregulated by *TBL1x* knockdown in both WT and NE-KO SMCs (Figure 5F), suggesting that MECP2 is negatively regulated by TBL1x. Importantly, the BioGRID (Biological General Repository for Interaction Datasets) network (https://thebiogrid.org/112770) predicts a close interaction between TBL1x, MECP2, and HDAC3 (Figure S12), suggesting a regulatory role for HDAC3 in TBL1x-mediated gene regulation. Indeed, data from chromatin immunoprecipitation assays showed that while increased binding of TBL1x and HDAC3 to the *MECP2* gene promoter was observed in NE-KO SMCs, *TBL1x* knockdown significantly inhibited this binding (Figure 5G and 5H, right). A similar effect was also observed in TBL1x and HDAC3 binding to the *LTA4H* gene promoter in THP-1 monocytes (Figure 5G and 5H, left). These data collectively confirm a regulatory role for TBL1x/HDAC3/MECP2 signaling in NE-mediated SMC phenotypic modulation under TAD pathological conditions.

### Tbl1x Inhibition Abrogates the Protective Effect of NE Gene Deficiency on BAPN-Induced Aortic Dissection Formation

**Figure 6.**
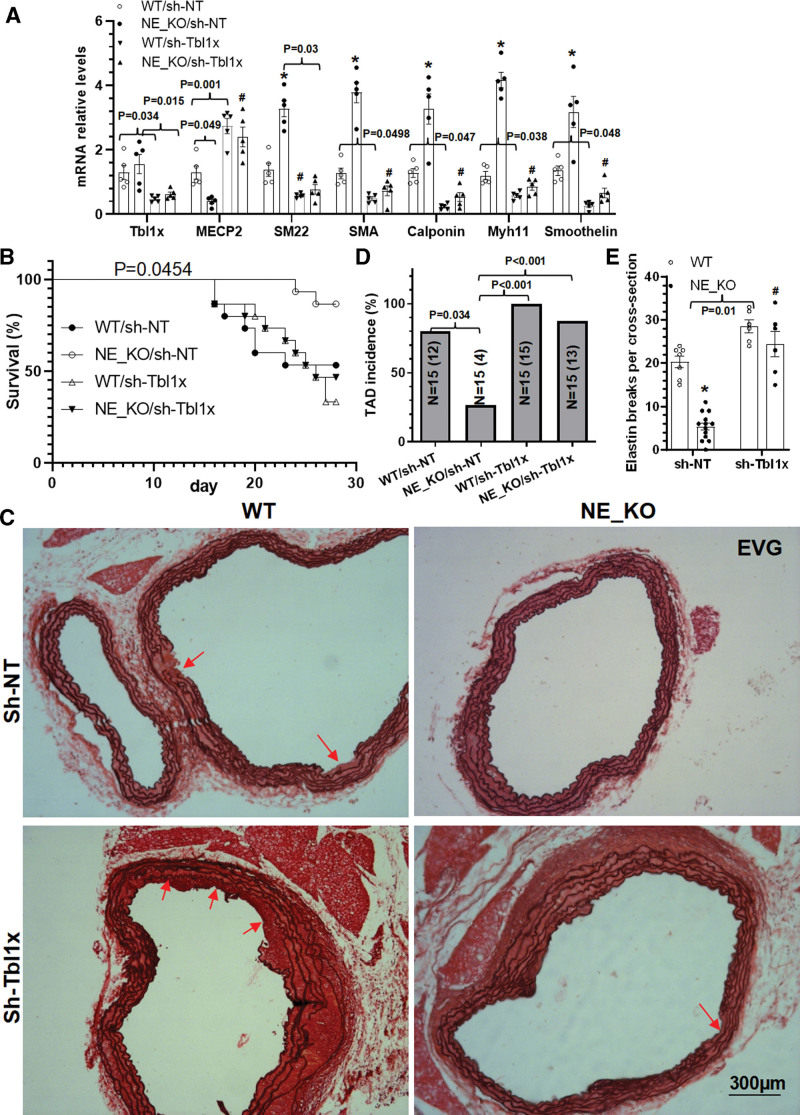
**Tbl1x (F-box-like/WD repeat-containing protein TBL1x) inhibition abrogates the protective effect of NE (neutrophil elastase) gene deficiency on β-aminopropionitrile monofumarate (BAPN)–induced AD formation.** Three-week-old sex-matched *ApoE*^−/−^/*NE*^+/+^ (WT) or *ApoE*^−/−^/*NE*^−/−^ (NE-KO) mice were randomly injected with a nontarget control (sh-NT [non-targeting shRNA]) or *Tbl1x* gene-specific (sh-Tbl1x) small hairpin RNA (shRNA) adeno-associated virus-2 (AAV2), followed by BAPN administration as shown in Figure S13A. Thoracic aortas were collected at 1 (**A**) or 4 (**C–E**) weeks, respectively, followed by various analysis. **A**, Aortic gene expression levels detected by real-time quantitative polymerase chain reaction (RT-qPCR) analysis. **B**, Animal survival curves (n=15 mice per group). **C** through **E**, Representative images for elastin van Gieson (EVG) staining (**C**), and the quantitative data of thoracic aortic dissection (TAD) incidence (**D**, 12/15 indicates 12 of 15 mice have TAD) and elastin breaks (**E**) were included here. Red arrows indicate TAD or intima tear. Data presented here are representative (**C**) or mean±SEM of 5 (**A**; n=5) or 6 to 13 mice (**C–E**, n=6–13 mice), respectively. *P<0.001 (versus WT) and #*P*<0.001 (versus sh-NT); if any analysis with *P*<0.05, but *P*>0.001, the exact *P* value was included in respective figures. **A** and **E**, Two-way ANOVA with a post hoc test of Tukey analysis. **B**, Log-rank (Mantel-Cox) test. **D**, χ^2^ test.

To further explore a potential role for TBL1x in NE-mediated SMC phenotypic modulation and TAD, an in vivo study was performed in WT and NE-KO mice using AAV2 vectors for specific gene transfer and knockdown in aortic SMCs as described previously^20^ and shown in Figure S13 and S14. Data from gene expression profiles revealed that while *MECP2* gene expression was significantly declined in NE-KO aorta, *Tbl1x* gene inhibition dramatically upregulated *MECP2* gene expression in both WT and NE-KO mice. Importantly, an opposite trend was observed with all the SMC genes (Figure 6A), confirming a regulatory role for TBL1x in *MECP2* and SMC gene expression in the context of TAD pathologies. Phenotypically, WT mice that received AAV2-sh-Tbl1x injection displayed a much higher mortality rate, while a comparable level of mortality was observed in WT/AAV2-sh-NT and NE-KO/AAV2-sh-Tbl1x mice (Figure 6B). Moreover, we observed a lower TAD incidence and decreased levels of elastic fiber breaks in NE-KO mice when injected with AAV2-sh-NT but a higher level of TAD incidence and elastic fiber breaks in both WT and NE-KO mice with *Tbl1x* gene silencing (Figure 6C through 6E; Figure S15). These data have collectively demonstrated that modulation of TBL1x/MECP2 signaling is one of the main underlying mechanisms of NE-mediated TAD formation.

### Increased NE Was Observed in Patients With Acute TAD

**Figure 7.**
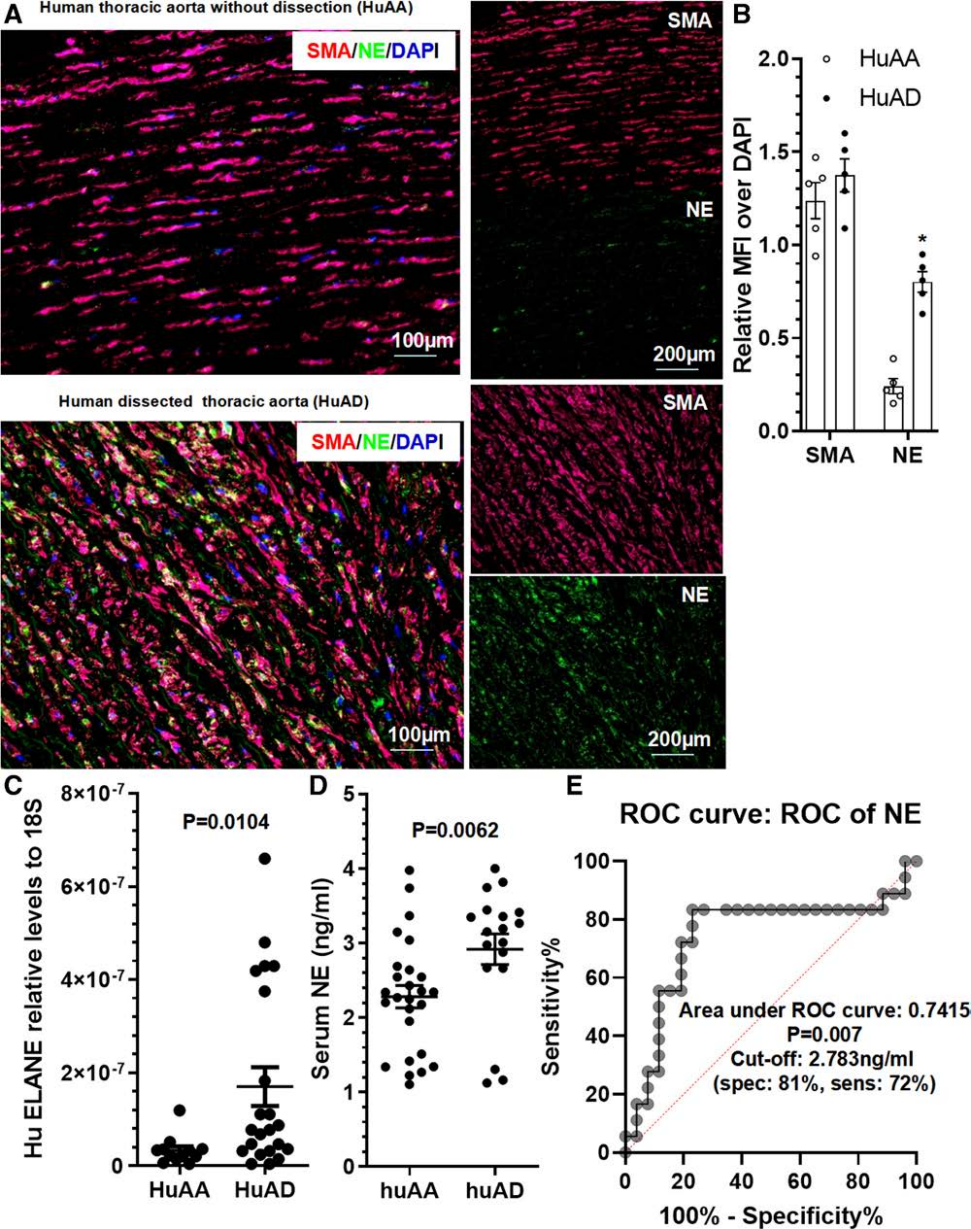
**NE (neutrophil elastase) detection in human thoracic aorta with (HuAD) or without (HuAA) acute thoracic aortic dissection (TAD). A** and **B**, Immunofluorescence (IF) staining showed increased expression levels of NE protein in dissected human thoracic aorta. Representative images (**A**) and relative mean fluorescence intensity (MFI; **B**) of NE or SMA (smooth muscle actin) over DAPI (4,6-diamidino-2-phenylindole) staining are presented here. *P<0.001 (n=5, vs HuAA; unpaired *t* test). **C**, Real-time quantitative polymerase chain reaction (RT-qPCR) analysis showed increased NE gene expression in human thoracic aorta with dissection. *P*=0.0104 (n=12 for HuAA or n=22 for HuAD vs HuAA; Mann-Whitney *U* test). **D**, ELISA analysis showed increased serum NE levels in patients with acute TAD. *P*=0.0062 (versus HuAA, n=26 for HuAA and n=18 for HuAD; Mann-Whitney *U* test). **E**, Receiver operating characteristic (ROC) curve showing the diagnostic ability of serum NE levels in human acute TAD.

We have so far demonstrated a critical role for NE in AA and dissection onset and aortic rupture in mice. To translate our findings from mice to patients, we examined NE gene expression in human arteries with or without acute TAD collected in our previous study.^24^ Data from H&E (hematoxylin and eosin) and elastin van Gieson staining confirmed TAD pathological characteristics including disordered elastic lamellae with frequent elastin breaks, depletion of elastic fibers, and SMC loss in the dissected human arteries, while no such pathological characteristics were observed with normal, healthy aorta (Figure S16). IF staining with antibodies against SMA and NE showed that NE was highly expressed in SMCs within the dissected human aorta (Figure 7A) and that NE protein expression was significantly higher in the dissected arteries compared with normal healthy human arteries (Figure 7A and 7B). Similarly, increased aortic NE gene expression was also observed in patients with acute TAD (Figure 7C). Additionally, we observed increased serum NE level in patients with acute TAD (Figure 7D), supporting a potential involvement of NE in the pathogenesis of human TAD. Importantly, a receiver operating characteristic curve showed a good diagnostic ability of serum NE levels in human acute TAD as evidenced by an area under the receiver operating characteristic curve of 0.7415 (Figure 7E). Specifically, by setting the serum NE level cutoff value at 2.783 ng/mL, we observed 81% specificity and 72% sensitivity for differentiation of the acute TAD patients from normal, healthy individuals (Figure 7E).

## DISCUSSION

TAD is a life-threatening medical emergency with high mortality and morbidity due to aortic rupture. Currently, no effective medical treatment is available to delay TAD onset and prevent aortic rupture due to a lack of novel insights into the etiology of TAD. Here, we demonstrate that NE plays a critical role in promoting TAD formation and progression. Specifically, genetic deletion and pharmacological inhibition of NE significantly decreased TAD incidence and reduced mortality by preventing aortic rupture, making NE a valuable therapeutic for treating patients at high risk of TAD onset and aortic rupture.

Multiple murine models have been established to study the etiology of TAD with a variable TAD incidence and mortality rate.^50,51^ In this study, we administrated 0.25% BAPN (wt/vol) into 3-week-old mice in drinking water and observed an 85% of TAD incidence and 46.7% of mortality rate, respectively. Our findings are consistent with observations reported in various previous studies^19–23^ but are slightly different from some observations reported in another study.^26^ Qi et al^26^ reported a 100% and 50% TAD incidence and mortality rate, respectively. Such a discrepancy may be attributable to differences in experimental settings and parameters used for evaluating TAD occurrence in these studies. For instance, mice used in our study are 3 weeks old, while adult mice (10–15 weeks of age) were used in their study. Additionally, although the mice used in both studies are on the same C57BL/6J genetic background, the mice used in our study also have ApoE gene deletion, which is a well-known influence factor for multiple aortic pathologies. Importantly, while coadministration of BAPN and Ang II into mice was used for inducing AA/aortic dissection by Qi et al, we administrated BAPN alone into mice to trigger TAD. Finally, the TAD incidence was defined as the mice that died from thoracic aortic rupture and the mice identified with ≥1 aortic pathologies (aortic intima tear, penetrating aortic ulcer, false lumen, and intramural hematoma, which was deemed when red blood cells or thrombi were found between elastic laminae) in our study, while the mice displayed additional major aortic pathologies including the widening of the interlaminar space, medial thinning and diminishment and the thickening of the tunic adventitia were also counted as AA/aortic dissection in the study by Qi et al. It is worth mentioning that we might miss some TAD cases where dissections were not grossly obvious due to resolution of intramural hematoma, which represents one of the limitations in our study.

We have previously reported a critical role for NE in atherosclerosis^16^ and injury-induced neointima formation.^18^ Specifically, we found that NE promotes atherosclerosis and increases plaque vulnerability by increasing systemic and aortic inflammation. NE exerts its proinflammatory role through increasing ATP-binding cassette transporter ABCA1 protein degradation and inhibiting macrophage cholesterol efflux, thereby facilitating foam cell formation.^16^ Moreover, a functional role for NE in injury-induced neointima formation and a therapeutic potential based on targeting NE in treating restenosis after angioplasty has been recently established using multiple approaches. Our study showed that NE promotes SMC proliferation, migration, and inflammation, and NE mediates these SMC dysfunctional phenotypes and injury-induced neointimal SMC hyperplasia through regulating the TLR4 (toll-like receptor 4)/MyD88 (myeloid differentiation primary response protein 88)/IRAK1 (interleukin-1 receptor–associated kinase 1)/TRAF6 (TNF receptor–associated factor 6)/NF-κB (nuclear factor kappa B) regulatory axis.^18^ Interestingly, Eliason et al^52^ were the first to report that circulating neutrophils and their infiltration into the aortic wall are an important initial trigger for experimental AA formation induced by aortic elastase perfusion, and neutrophil depletion inhibits AA development, indicating an important role for neutrophils in AA formation. Additionally, DPPI (dipeptidyl peptidase I)—a lysosomal cysteine protease that is critical for activating neutrophil granule-stored serine proteases including NE—was reported to play an essential role in the development of elastase-induced experimental AA by promoting neutrophil recruitment to the elastase-injured aortic wall and increasing local production of the CXC chemokine ligand 2.^53^ Importantly, a later study from the same research group showed that the absence of 2 neutrophil serine proteases (NE and proteinase-3) could recapitulate the AA-resistant phenotype of DPPI-deficient mice,^54^ inferring an important role for NE in AA development. Moreover, drug inhibition of elastase activity prevented elastase infusion–induced experimental abdominal AA progression.^55^ Concerning a potential involvement of inflammatory cells in TAD, it has been reported that targeted depletion of monocytes/macrophages in mice inhibited the occurrence of TAD,^56^ confirming a critical role for these inflammatory cells in TAD development. Moreover, intravenous administration of an NE inhibitor sivelestat during surgery restored the postoperative antithrombin III and platelet levels in patients undergoing emergency surgery for acute TAD. This intervention also shortened the duration of mechanical ventilation after surgery,^57^ suggesting a beneficial effect for NE inhibition for acute TAD patients undergoing emergency surgery. However, the abovementioned studies mainly used an elastase infusion–induced experimental AA to study the potential involvement of neutrophil serine proteases in this aortic disease, which somehow impedes our understanding into the causal effect of endogenous NE on AA formation and aortic dissection onset/progression. In this study, we first found that NE expression and activity were increased in BAPN-induced TAD and patients with acute TAD. Importantly, data from both genetic deletion and pharmacological inhibition experiments confirmed a causal role for endogenous NE in TAD onset and progression, as well as aortic rupture, and demonstrated convincingly a therapeutic potential for NE inhibitors in treating patients with TAD.

Interestingly, our data showed that apart from neutrophils, NE is also highly expressed in other cells including macrophages and SMCs in BAPN-induced TAD (Figures S7, S8, S9A, and S10) and dissected human arteries (Figure 7A), which is in line with our previous observations in other pathological conditions such as atherosclerotic plaques^16^ and injured arteries.^18^ NE was dramatically upregulated in aortic SMCs by Ang II and BAPN combined treatment, and it was produced and secreted by SMCs as evidenced by higher NE enzymatic activity detected in both cell lysate and cell culture supernatant (Figure S9B through S9D). These results suggest that in the in vivo disease setting of TAD, multiple cells (eg, neutrophils, macrophages, and SMCs) can interact with and regulate each other’s functions through an autocrine or paracrine manner. Indeed, we observed that a large number of NE-expressing neutrophils were recruited/migrated to the media layer and dissection sites (Figure S7). We, therefore, speculate that these recruited inflammatory cells, as well as activated resident SMCs, produced/secreted NE, which directly cleaves/degrades ECM proteins (eg, collagens, elastin, glycoproteins), weakening aortic wall. On the other hand, produced/secreted NE may also act on SMCs and cause SMC dysregulation, thereby promoting TAD onset. However, it is worth mentioning that one of the limitations in this study is that our current data do not allow us to comprehensively elaborate on the potential for TAD formation from different cellular NE sources. Moreover, the nature of NE as a secretory protein makes it extremely difficult to achieve such a delineation, since this type of protein can exert its cellular functions in both an autocrine and paracrine manner. For instance, we have previously reported that adventitia stem/progenitor cells do not express MMP-8 but instead uptake MMP-8 secreted by macrophages, which in turn promotes their differentiation toward SMCs, thus contributing to injury-induced neointima formation.^58^

Mechanistically, we have provided clear evidence to support the idea that modulating the formation of NETs (NETosis) under TAD pathological conditions is one of the molecular mechanisms underpinning NE-mediated TAD formation/progression. NETosis is positively associated with increased AA expansion rates^59^ and has been identified as a novel biomarker and therapeutic target to inhibit the progression of AA.^60^ NETosis has been found to occur at early stages of experimental AA (as early as 2–3 days after aneurysm induction),^54,61^ and it has been widely reported as one of the key driving causes and determinants of AA formation and development.^54,61–63^ Importantly, using DNase I to degrade and inhibit NETosis, a therapeutic potential based on targeting NETosis in elastase-induced experimental AA was successfully confirmed.^53,54^ Consistent with these observations, we observed abundant NETosis in WT but not in NE-KO aorta and neutrophils under TAD pathological conditions in this study, which could attribute to the TAD-resistant phenotype observed in NE-deficient mice and WT mice that received NE inhibition treatment.

Both MMP-2 and MMP-9 have well-established causal roles in TAD pathogenesis.^64^ Specifically, using compound-deficient mice and macrophage infusion, researchers were able to show that macrophage-derived MMP-9 and mesenchymal cell MMP-2 are both required and work in concert to produce AA.^65^ Further study showed that neutrophil-derived MMP-9 is an important trigger for acute TAD.^66^ Interestingly, multiple studies have demonstrated a regulatory role for NE in regulating and activating both MMP-2^67^ and MMP-9^68^ under various experimental conditions. NE has been suggested as a physiological activator of MMP-2 and MMP-9 in tumor invasion and angiogenesis,^67^ acute lung injury,^68^ or during extended culture of monocytes and fibroblasts in 3-dimensional collagen gels.^69^ Importantly, apart from activating MMPs, NE has also been reported to upregulate MMP-2 expression^70^ and increase production of active MMPs,^71^ thereby activating NF-κB signaling in macrophages. Moreover, it has been reported that increased NE activity could compensate for *MMP-9* gene inactivation in regulation of leukocyte infiltration in a model of experimental peritonitis.^72^ Furthermore, NE has also been implicated in human cystic fibrosis proteolytic dysfunction as evidenced by its ability to discretely cleave and activate MMP-9 but degrade the tissue inhibitor of metalloprotease-1.^73^ The aforementioned findings are nicely aligned with our observation herein that both the expression levels and activities of MMP-2 and MMP-9 were significantly decreased in NE-KO aorta under BAPN treatment (Figure S5). Therefore, it is plausible for us to conclude that increasing MMP-2/9 expression or activity in the aorta in response to BAPN treatment is another underlying molecular mechanism of NE-mediated TAD onset and development.

Moreover, one of the important and novel findings in the current study is that we have identified TBL1x as a functional NE substrate and target in the context of TAD and a key downstream signal molecule through which NE promotes TAD formation and progression. Specifically, we have provided several lines of evidence to support this conclusion. First, TBL1x was identified as the most significantly regulated protein in NE-KO aorta in response to Ang II infusion in our aortic proteomics analysis. Second, we found a significant increase of aortic TBL1x protein but not its mRNA expression level in NE-KO mice upon BAPN treatment. Third, data from our in vitro protein digestion provide direct evidence to show that TBL1x protein can be directly cleaved and degraded by NE. Further multiple biochemical and biological assays confirmed TBL1x is a functional downstream target of NE in the context of inflammatory cell transendothelium migration and vascular SMC phenotypic modulation. Finally, but importantly, the TAD-resistant phenotype observed in NE-deficient mice disappeared following AAV2-mediated Tbl1x gene inhibition in aortic SMCs under BAPN treatment, suggesting a critical role for TBL1x in NE-mediated TAD onset and progression.

TBL1x and HDAC3 were initially identified as 2 key components of the core SMRT (silencing mediator of retinoid and thyroid hormone receptors) corepressor complex.^74,75^ A later study showed that TBL1x is required for transcriptional activation mediated by liganded nuclear receptors and PPARγ (peroxisome proliferator-activated receptor gamma)-induced adipogenic differentiation from embryonic stem cells.^76^ Moreover, TBL1x was found to play a regulatory role in Wnt (wingless-type MMTV integration site family)-β-catenin signaling either by directly interacting with and recruiting β-catenin into nuclei^77^ or protecting β-catenin from Siah-1 (seven in absentia homolog) ubiquitination.^78^ A similar role was reported for TBL1x in regulating NF-κB signaling in breast cancer cells.^79^ Importantly, it has been shown that MECP2 directly binds to TBL1x and its related protein TBLR1, and such interaction is crucial for optimal brain function.^80^ Interestingly, we have previously demonstrated that MECP2 is a key suppressor of SMC differentiation^49^ and phenotypic modulation.^36^ Accordingly, we speculated that MECP2 might play a regulatory role in TBL1x-mediated SMC phenotypic modulation. Indeed, we found that *MECP2* gene expression was negatively regulated by TBL1x during Ang II/BAPN-induced SMC phenotypic switching, and our findings confirmed that TBL1x could directly bind to *MECP2* gene promoter. Therefore, we provide clear evidence in this study to support the notion that TBL1x maintains the SMC contractile phenotype under TAD pathological conditions by suppressing MECP2 expression in SMCs.

It worth noting that the potential impact of NE inhibition and gene inactivation on TAD formation and onset may not be solely attributed to degradation of TBL1x and matrix fibers, and the underlying molecular mechanisms of NE-mediated TAD development may not be limited to those examined in the current study. As shown in Figure 3A and Figure S6, there are additional 154 proteins that were found to be modulated by *NE* gene inactivation in aorta. These modulated proteins, therefore, may represent other interesting targets through which NE promotes TAD onset and progression. Among them, IRGM1 (immunity-related GTPase family M1), CD180, FBN1 (fibrillin 1), and THSD4 (thrombospondin type 1 domain containing 4) are particularly interesting. Specifically, several genetic studies have identified multiple pathogenic variants in *THSD4* and *FBN1* gene and showed a causal association of these pathogenic variants with AA/TAD, but not with other vascular diseases,^81–83^ pinpointing a functional role for these 2 proteins in AA/TAD. Moreover, CD180, the toll-like receptor protein RP105, has been identified to mediate the innate immune response and NF-κB activation in cooperation with TLR4.^84^ Interestingly, we have previously identified TLR4 as the functional target of NE in mediating aortic inflammation,^18^ inferring a potential role for CD180/TLR4 signaling in NE-mediated aortic inflammation and TAD development. Furthermore, IRGM1 has been widely reported to regulate inflammatory cell motility,^85^ macrophage polarization,^86^ and autoimmunity,^87^ all of them could contribute to TAD formation. Therefore, the functional implications of these proteins in NE-mediated TAD onset and progression warrant further investigation.

In conclusion, we have uncovered a critical role for NE in TAD formation/progression and have demonstrated that NE promotes TAD onset and aortic rupture by enhancing inflammatory cell recruitment/migration into vascular walls, increasing aortic inflammation, and facilitating SMC phenotypic switching. NE exerts the abovementioned cellular functions at least partially through directly cleaving and degrading TBL1x protein, which desuppresses LTA4H signaling and subsequently enhances inflammatory cell transendothelium migration, thereby increasing aortic inflammation. Simultaneously, decreased TBL1x also desuppresses MECP2 expression, which in turn promotes SMC switching toward a dedifferentiated phenotype. TBL1x represses both MECP2 and LTA4H expression through increasing direct binding of HDAC3 to the *MECP2/LTA4H* gene promoter. As reported previously,^18^ NE also increases aortic and SMC inflammation through modulating the TLR4/NF-κB signaling pathway. Importantly, NE can also directly upregulate and activate both MMP-2 and MMP-9 and promote NETosis. Therefore, NE promotes TAD formation through multiple signaling pathways including, but not limited to, MMP-2/9 activation, aortic inflammation, SMC switching into synthetic phenotype, and NETosis. Importantly, this study also highlights the therapeutic potential of NE inhibition in patients with TAD.

## ARTICLE INFORMATION

### Sources of Funding

This work was partially supported by National Key R&D Program of China (2022YFA1104200), the British Heart Foundation (PG/15/11/31279, PG/15/86/31723, PG/16/1/31892, PG/20/10458, and PG/23/11371), National Natural Sciences Foundation of China (82125005, 82100503, 81800364, 81930010, and 81870206), and Shanghai Shenkang Three-Year Action Plan for Promoting Clinical Skills and Clinical Innovation in Municipal Hospitals (SHDC2020CR4015). This work forms part of the research portfolio for the National Institute for Health Research Biomedical Research Centre at Barts.

### Disclosures

None.

### Supplemental Material

Table S1

Figures S1–S16

Major Resources Table

Full Unedited Blots

## Supplementary Material
