# Supplementary material for: Causal Role for Neutrophil Elastase in Thoracic Aortic Dissection in Mice
Source: Arterioscler Thromb Vasc Biol. 2023 Aug 17;43(10):1900–20. doi: 10.1161/ATVBAHA.123.319281 (PMC10521802; doi:10.1161/ATVBAHA.123.319281)
Supplement: Supplementary file 1 [file atv-43-1900-s001.doc]

***SUPPLEMENTAL MATERIALS***

**Causal Role for Neutrophil Elastase in Thoracic Aortic Dissection in Mice**

(Yang et al. NE in aortic dissection)

**Mei Yang^1,2#^, Xinmiao Zhou^2,3#^, Stuart W.A. Pearce^2#^, Zhisheng Yang^2^, Qishan Chen^1^, Kaiyuan Niu^2^, Chenxin Liu^2^, Jun Luo^4^, Dan Li^1,5^, Yue Shao^4^, Cheng Zhang^4^, Dan Chen^4^, Qingchen Wu^4^, Pedro R Cutillas^6^, Lin Zhao^5*†^, Qingzhong Xiao^2,7*†^, and Li Zhang^1*†^**

^1^Department of Cardiology, and Institute for Developmental and Regenerative Cardiovascular Medicine, Xinhua Hospital Affiliated to Shanghai Jiaotong University School of Medicine, Shanghai, 200092, China;

^2^William Harvey Research Institute, Faculty of Medicine and Dentistry, Queen Mary University of London, London EC1M 6BQ, UK;

^3^Department of Respiratory and Critical Care Medicine, Sir Run Run Shaw Hospital, Zhejiang University School of Medicine, Hangzhou, China.

^4^Department of Cardiothoracic Surgery, The First Affiliated Hospital of Chongqing Medical University, Chongqing, PR China;

^5^Department of Cardiology, Beijing Anzhen Hospital, Capital Medical University, Beijing, China

^6^Centre for Haemato-Oncology, Barts Cancer Institute, Faculty of Medicine and Dentistry, Queen Mary University of London, United Kingdom;

^7^Key Laboratory of Cardiovascular diseases, School of Basic Medical Sciences; Guangzhou Institute of Cardiovascular Disease, The Second Affiliated Hospital, Guangzhou Medical University, Guangzhou 511436, China

**Supplementary table S1**: Primer sets used in the present study (Mus, Mouse; Hu, human)

| **Gene names** | **Forward (5’-3’)** | **Reverse (5’-3’)** | **Application** |
| --- | --- | --- | --- |
| 18s (mus/hu) | AAACGGCTACCACATCCAAG | CCTCCAATGGATCCTCGTTA | RT-qPCR |
| Mus NE | CAGAGGCGTGGAGGTCATTT | CTACCTGCACTGACCGGAAA | RT-qPCR |
| Hu NE | CGTGGCGAATGTAAACGTCC | TTTTCGAAGATGCGCTGCAC | RT-qPCR |
| Mus TBL1x | CCACAAGTTGCACGGCTC | GTGAGCCACCCTCGTCAC | RT-qPCR |
| Mus LTA4H | GGGGCACATAAAGCGAATGC | GCAGAGCCGTAACCATCTGA | RT-qPCR |
| Mus MCP1 | CCCCAAGAAGGAATGGGTCC | TGCTTGAGGTGGTTGTGGAA | RT-qPCR |
| Mus IL6 | GTGGCTAAGGACCAAGACCA | TAACGCACTAGGTTTGCCGA | RT-qPCR |
| Mus IL12β | AGTGACATGTGGAATGGCGT | CAGTTCAATGGGCAGGGTCT | RT-qPCR |
| Mus VCAM1 | TTCTGACGTGTGCTGCTATTGG | TTTGGCCCCCTCATTCCTT | RT-qPCR |
| Mus ICAM1 | GTGGGTCGAAGGTGGTTCTT | AAACAGGAACTTTCCCGCCA | RT-qPCR |
| Mus MECP2 | GGCTGTGGTAAAACCCGTCCG | GGCTTGTCTCTGAGGCCCTGGA | RT-qPCR |
| Mus SMαA | TCCTGACGCTGAAGTATCCGAT | GGCCACACGAAGCTCGTTATAG | RT-qPCR |
| Mus SM22α | GATATGGCAGCAGTGCAGAG’ | AGTTGGCTGTCTGTGAAGTC | RT-qPCR |
| Mus Calponin | GGTCCTGCCTACGGCTTGTC | TCGCAAAGAATGATCCCGTC | RT-qPCR |
| Mus Myh11 | AAGCAGCCAGCATCAAGGAG | AGCTCTGCCATGTCCTCCAC | RT-qPCR |
| Mus Smoothelin | GGGCAGTATCTTCGACCGAG | GGCAGACTCTGTGCCTTCAT | RT-qPCR |
| Mus TBL1x | TGGAACCATCTCCACATCTGC | ACCCGTTTGTCTAGGGGGTA | ChIP assay |
| Hu LTA4H | GCCCTCAACAAACTTACGCC | GGACACAGCTAATGGAACGC | ChIP assay |

**Supplementary Figures:**

**Figure S1. Aortic pathologies of WT and NE_KO mice at baseline.**

Thoracic (TA) and abdominal (AA) aortas were collected from eight week-old WT or NE_KO mice and subjected to HE staining. Data presented here are representatives (A) or Mean±S.E.M (B) of five mice (n=5 mice).

**Figure S2. BAPN-induced TAD formation was comparable in male and female mice.**

Three week-old male or female mice were fed a normal diet and administered with freshly prepared 3-aminopropionitrile fumarate (BAPN) solution dissolved in the drinking water (0.25% wet/vol) for four weeks. (A) Kaplan–Meier survival curve showing animal survival rate. Note: 4/10 indicates 4 out of 10 mice dead. P=0.52 (n=10 mice per group, log-rank (mantel-cox) test). The quantitative data of TAD incidence (B, 9/10 indicates that 9 out of the 10 mice have TAD; p=0.5318, Chi-square test, n=10 mice per group) and elastin breaks (C, p=0.8433, unpaired *t*-test, n=10 mice per group) were presented here. (D) Thoracic aortic NE gene expressions in male and female mice (p=0.718, unpaired *t*-test, n=10 mice per group).

**Figure S3. Increased NE expression levels and activity during BAPN-induced TAD formation.**

Three-week old age and sex (male: female=2:1) matched mice were fed a normal diet and randomly administered with vehicle (water) or freshly prepared BAPN solution dissolved in the drinking water (0.25%, wt/vol) for indicated times, thoracic aortas and plasma were collected and subjected to histological (A-C), RT-qPCR (D), and NE activity (E) analysis, respectively. Representative images of HE (A), Elastin Van Gieson (EVG) staining (B-C), and quantitative data of aortic NE expression (D) and plasma NE activity (F) from five mice (n=5 mice) were presented here. Red arrow indicates TAD site. A higher magnification of images within green boxes were included (bottom) in (B). Blue arrow indicates intramural hematoma in (C). Exact P values were included in B-D (versus vehicle, one-way ANOVA with a post hoc test of Tukey’s analysis).

**Figure S4.** **Decreased NETs were observed in dissected aortas from NE_KO mice and NE-deficient neutrophils**.

(A-B) Three-week old WT or NE_KO mice were administered with BAPN in drinking water for two weeks. Thoracic aortas were collected and subjected to immunofluorescence (IF) staining with the indicated antibodies against NETosis-associated proteins (Cit-H3, Citrullinated histone H3; MPO, myeloperoxidase). (C-D) Neutrophils isolated from WT or NE_KO mice were treated with Phorbol-1-myristate-13-acetate (PMA, 100 nM) to induce NETosis. Cells were fixed and subjected to IF staining with the indicated antibodies. Representative images (A & C) and relative mean fluorescence intensity (MFI) of the target proteins over DAPI signal (B & D) from six mice (n=6) were presented here. Exact P values were included in B and D (versus WT, unpaired *t*-test).

**Figure S5. Reduced inflammatory cell accumulation and decreased MMP2/9 activity in NE_KO dissected aortas.**

Three-week old WT and NE_KO mice were administered with BAPN in drinking water for two weeks, thoracic aortic tissues were collected and subjected to IF staining (A-F), MMP-2/9 activity (G), and RT-qPCR (H) assays, respectively. (A-C) IF staining analysis of the Ly-6G^+^ neutrophils and MMP2 expression in thoracic aorta. (B) Percentage of Ly-6G^+^ neutrophils. (C) Quantitative analysis of MMP2 protein expression levels. (D-F) IF staining analysis of the Iba1^+^ macrophages and MMP9 expression in thoracic aorta. (E) Percentage of Iba1^+^ macrophages. (F) Quantitative analysis of MMP9 protein expression levels. Data presented here are representative (A & D) or Mean±S.E.M of ten mice (B, C, E, F, n=10 mice). (G) Thoracic aortic total and active MMP2 and MMP9 levels. Total and active MMP2 and MMP9 levels were assessed using the FRET peptide-based immunocapture assay. (H) RT-qPCR analysis of inflammatory gene expressions in thoracic aorta. Data presented in (G-H) are Mean±S.E.M of six mice (n=6 mice). Exact P values were included in B-H (versus WT, unpaired *t*-test).

**Figure S6. List of all the up-regulated and down-regulated proteins in NE_KO abdominal aorta in response to Ang II infusion for two weeks**.

**Figure S7. NE expressing neutrophils were observed in the dissected sites.**

Three-week old WT mice were administered with BAPN in drinking water for three weeks, thoracic aortic tissues were collected and subjected to IF staining. Data presented here are representative images from six mice (n=6 mice). Green arrows indicate NE expressing neutrophils, and red arrows indicate AD, respectively.

**Figure S8. Immunofluorescence (IF) staining showing macrophages express Tbl1x protein expression in aorta.**

Three-week old mice were administered with BAPN in drinking water for two weeks, thoracic aortic tissues were collected and subjected to IF staining analysis with the indicated antibodies. Representative images from five mice (n=5) were presented here. White arrows indicate TBL1x expressing macrophages.

**Figure S9. Ang II/BAPN co-stimulated NE expression in aortic SMCs**

(A) NE expression was up-regulated in thoracic aortic SMCs during BAPN-induced TAD development. (B-D) BAPN treatment increased Ang II-induced NE gene expression and activity in SMCs. Serum starved SMCs were treated with 10nM Ang II and increasing amount of BAPN for 24 hours. Total RNAs (B), cell lysate (C), and conditioned culture medium (D) were collected and subjected to RT-qPCR analysis (B) and NE activity assay (C-D), respectively. Data presented here are representative (A) of six mice (n=6) or Mean±S.E.M of five independent experiments (B-D, n=5), respectively. Exact P values were included in B-D (versus control, one-way ANOVA with a post hoc test of Tukey’s analysis).

**Figure S10. IF staining showing increased TBL1x protein expression in NE_KO aorta.**

Three-week old WT and NE_KO mice were administered with BAPN in drinking water for two weeks, thoracic aortic tissues were collected and subjected to IF staining analysis with the indicated antibodies. Representative images from five mice (n=5) were presented here. White arrows indicate TBL1x expressing aortic SMCs.

**Figure S11. Synergistic effect of Ang II and BAPN on SMC gene expression**

(A) RT-qPCR analysis of SMC genes in WT and NE_KO aortas treated with vehicle control (water) for two weeks. (B) Plasma Ang II. Three-week old WT or NE_KO mice were randomly administered with vehicle (water) or 0.25% BAPN (wt/vol) in drinking water for two weeks. Plasma Ang II levels were measured using a mouse angiotensin II ELISA kit (Enzo Life Sciences, Exeter, UK, ADI-900-204) as per manufacturer’s instructions. (C) SMC gene expression was not impaired by BAPN treatment. Serum starved SMCs were treated with increasing amount of BAPN for 24 hours. Total RNAs were collected and subjected to RT-qPCR analysis. (D) Synergistic effect of Ang II and BAPN on SMC gene expression. Serum starved SMCs were treated with vehicle control, 10 nM Ang II, or 10 nM Ang II plus 25 μg/ml BAPN for 24 hours. Total RNAs were collected and subjected to RT-qPCR analysis. Data presented here are Mean±S.E.M of five/six mice (n=5/6 mice, A/B) or five independent experiments (n=5, C/D), respectively. Exact P values were included in B and D (versus vehicle, two- (B) or one (D) way ANOVA with a post hoc test of Tukey’s analysis).

**Figure S12. TBL1x protein-protein interactions extracted and downloaded from BioGRID network (**[**https://thebiogrid.org/112770**](https://thebiogrid.org/112770)**).**

**Figure S13. AAV2-mediated *in vivo* gene transfection in aortic SMCs.**

(A) Experimental protocol for in vivo gene delivery, BAPN administration, and sample analysis. (B) At the end of protocol, thoracic aortic tissues were collected and subjected to IF staining analysis with antibodies against GFP and SMA. Representative images from five mice (n=5) were presented here.

**Figure S14. AAV2-sh-Tbl1x inhibits TBL1x protein expression in media SMCs.**

At the end of experiments as shown in Figure S13A, thoracic aortic tissues were collected and subjected to IF staining analysis with antibodies against TBL1x and SMA. Representative images (A) and semi-quantitative analysis of TBL1x and SMA protein expression in aortic media layer (B) or adventitia (C) from five mice (n=5) were presented here. Exact P values were included in B (versus sh-NT, unpaired *t*-test).

**Figure S15. Relate to Figure 6C (images with a high magnification of Figure 6C).**

**Figure S16.** **Representative images of H&E and EVG staining of human thoracic aorta with (HuAD) or without (HuAA) acute AD.**

**Major resource table**

Animal (in vivo studies)

| Species | Vendor or Source | Background Strain | sex | Persistent ID/URL |
| --- | --- | --- | --- | --- |
| ApoE^-/-^/NE^+/+^ | In house bred | C57BL/6 | M/F | None |
| ApoE^-/-^/NE^-/-^ | In house bred | C57BL/6 | M/F | None |

Genetically Modified Animals

|  | Species | Vendor or Source | Backgound Strain | Other information |
| --- | --- | --- | --- | --- |
| Parent Male | ApoE^-/-^/NE^+/+^ and ApoE^-/-^/NE^-/-^ | In house | C57BL/6 | None |
| Parent Female | ApoE^-/-^/NE^+/+^ and ApoE^-/-^/NE^-/-^ | In house | C57BL/6 | None |

Antibodies

| Target antigen | Vendor or Source | Catalog# | Working concentration |
| --- | --- | --- | --- |
| SMA | Abcam | ab5694 | 1μg/ml |
| SMA | Abcam | ab7817 | 1μg/ml |
| SM22 | Abcam | ab14106 | 1 µg/ml |
| NE | Abcam | ab68672 | 5μg/ml |
| CD68 | Abcam | ab125212 | 1 µg/ml |
| Iba1 | Abcam | ab15690 | 1:100 |
| Histone H3 (citrulline 2 + 8 + 17) | Abcam | ab5103 | 2.5μg/ml |
| HDAC3 | Abcam | ab137704 | 1:200 |
| LTA4H | Abcam | ab133512 | 1:10000 |
| MECP2 | Abcam | ab253197 | 1:1000 |
| MMP-9 | Abcam | ab58803 | 1:100 |
| MMP-2 | Abcam | ab37150 | 1:100 |
| SMA | Merck | A5228 | 1:2000 |
| α-Tubulin | Merck | T6074 | 1:5000 |
| TBL1x | Proteintech | 66955-1-Ig | 1:5000 |
| Ly6G | Biolegend | 127602 | 1:100 |
| myeloperoxidase (MPO) | R and D Systems | AF3667 | 5μg/ml |

Cultured Cells

| Name | Vendor or Source | Sex (F,M, or unknown) |
| --- | --- | --- |
| Mouse smooth muscle cells | In house isolated from murine aorta | Mixed sex |
