# Supplementary material for: Causal Role for Neutrophil Elastase in Thoracic Aortic Dissection in Mice
Source: Arterioscler Thromb Vasc Biol. 2023 Aug 17;43(10):1900–20. doi: 10.1161/ATVBAHA.123.319281 (PMC10521802; doi:10.1161/ATVBAHA.123.319281)

Full unedited blots for Figure 3B

Figure 3B_TBL1x

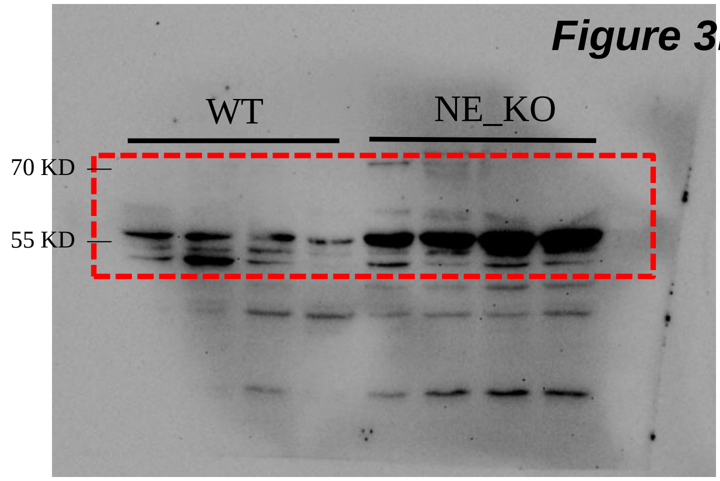

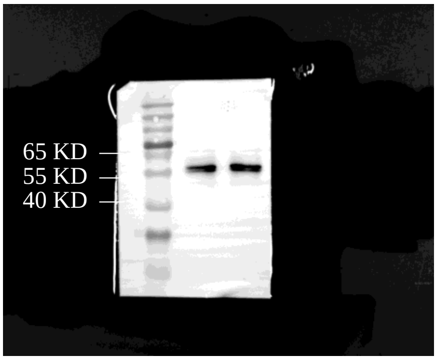

Figure 3B_LTA4H

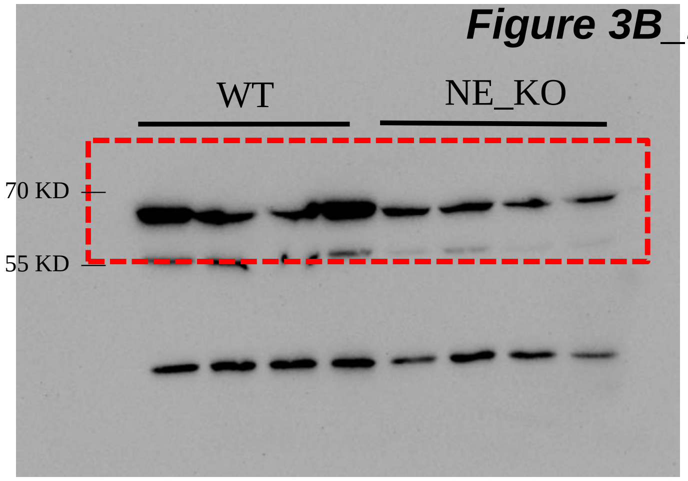

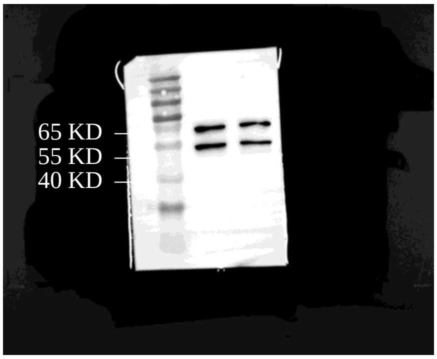

Figure 3B_Tubulin

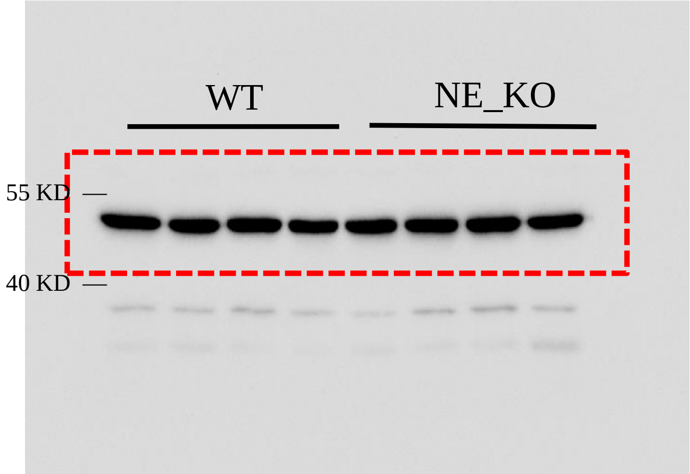

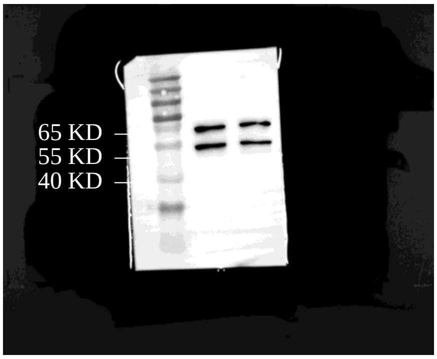

Full unedited blots for Figure 3E

Figure 3E_In vitro TBL1x protein digestion

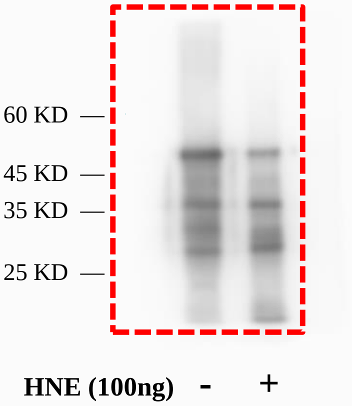

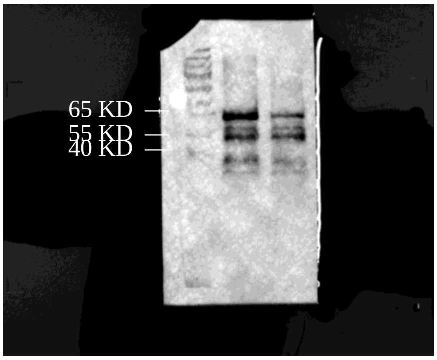

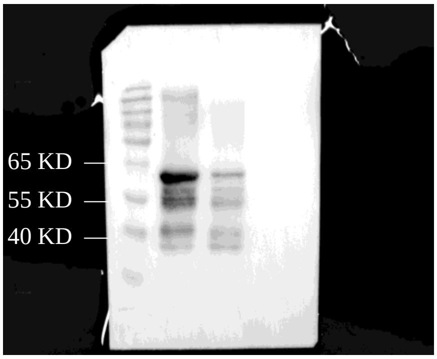

Figure 3E_In vitro LTA4H protein digestion

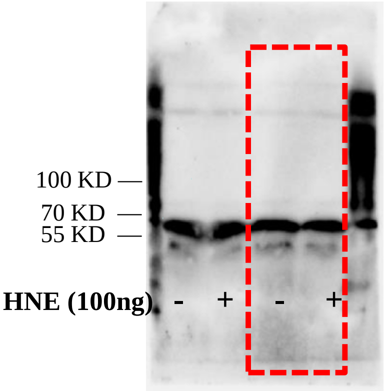

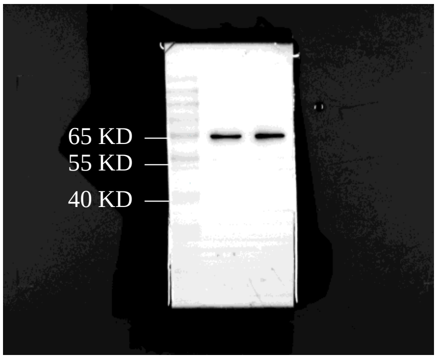

Full unedited blots for Figure 5B1

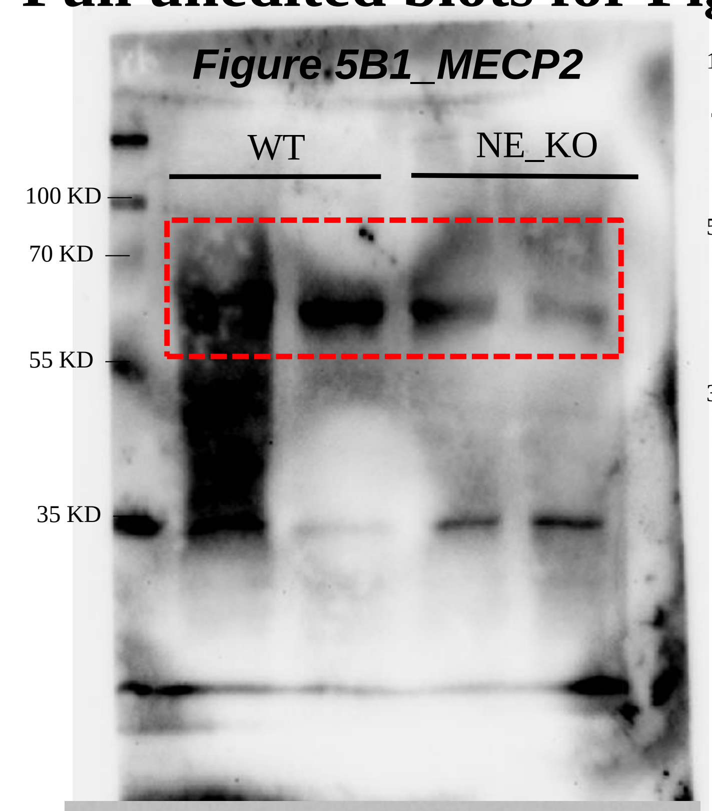

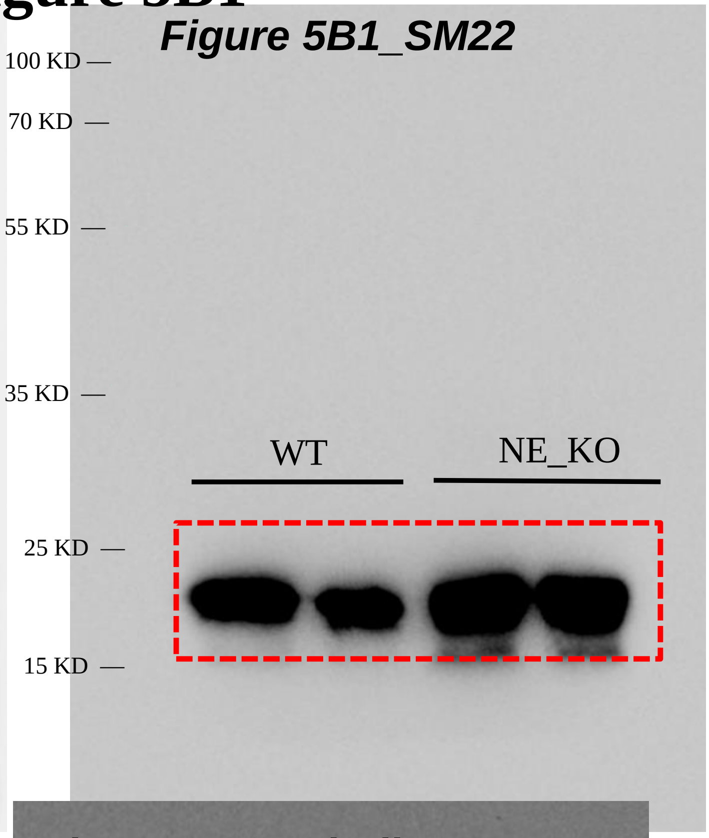

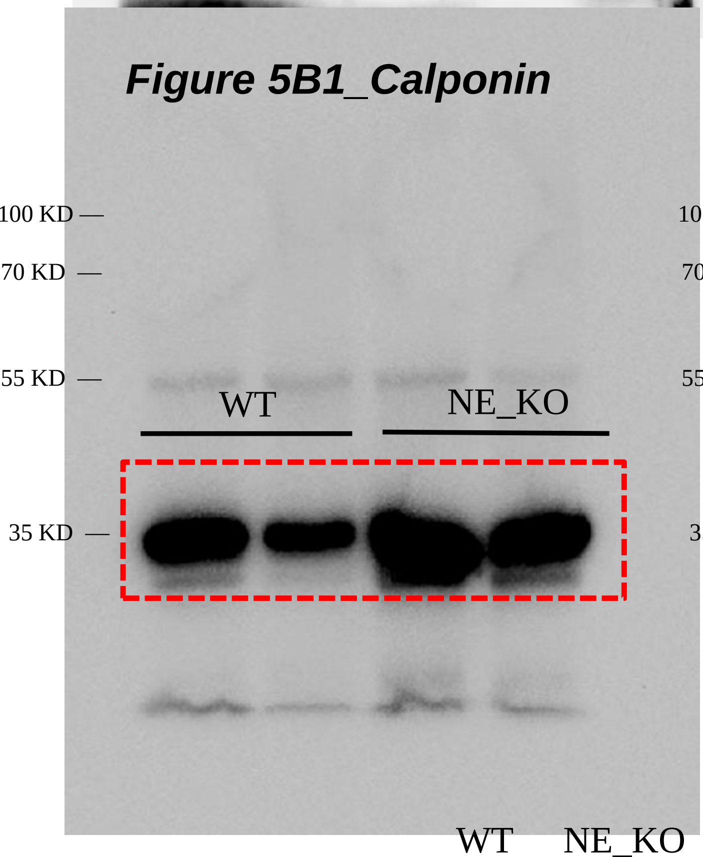

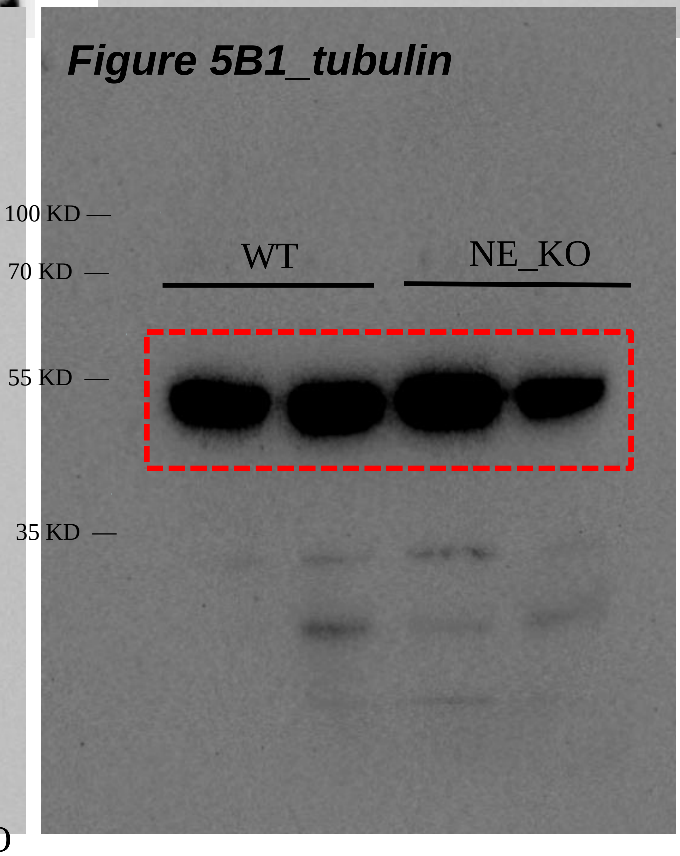

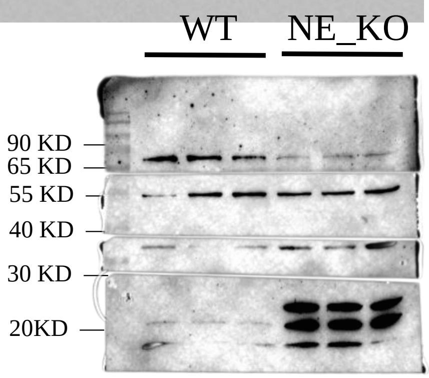

Figure 5B1

MECP2

Tubulin

calponin

sm22

Full unedited blots for Figure 5E1

Figure 5E1_TBL1x

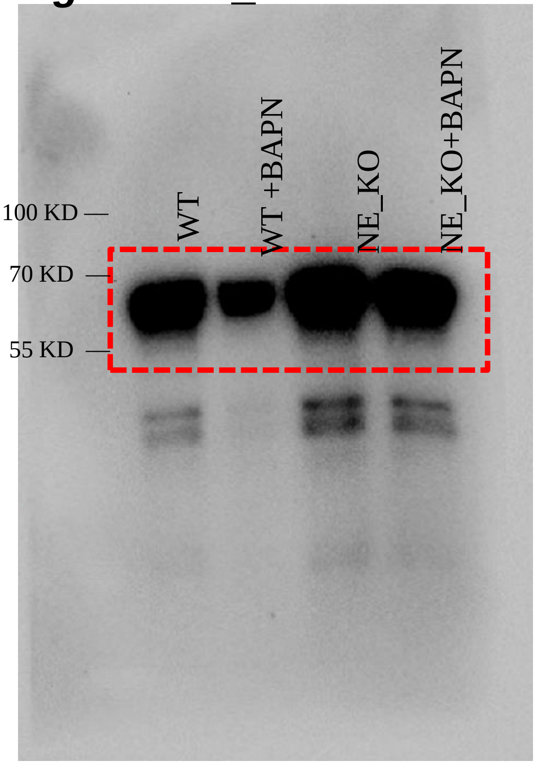

Figure 5E1_MECP2

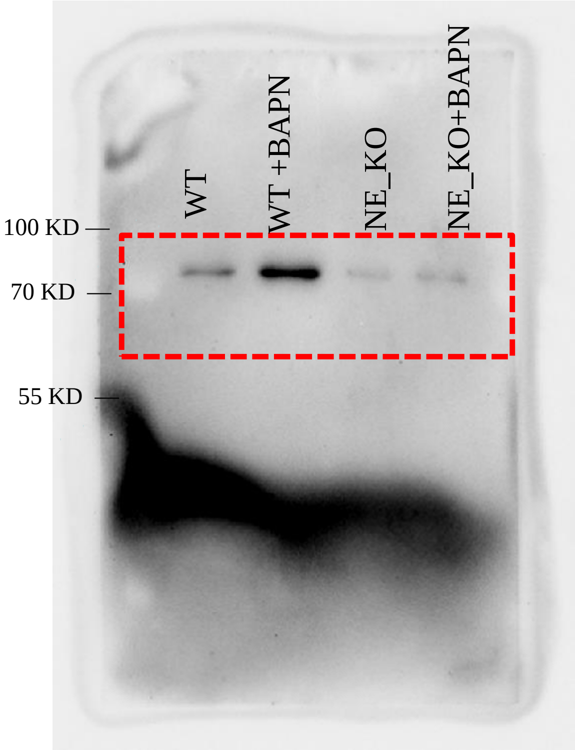

Figure 5E1_SMA

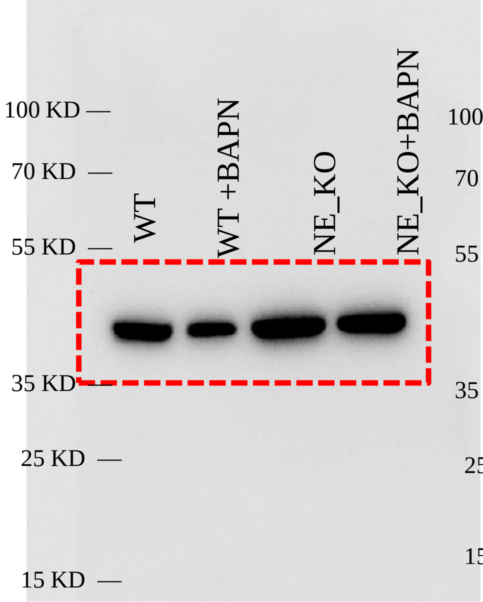

Figure 5E1_SM22

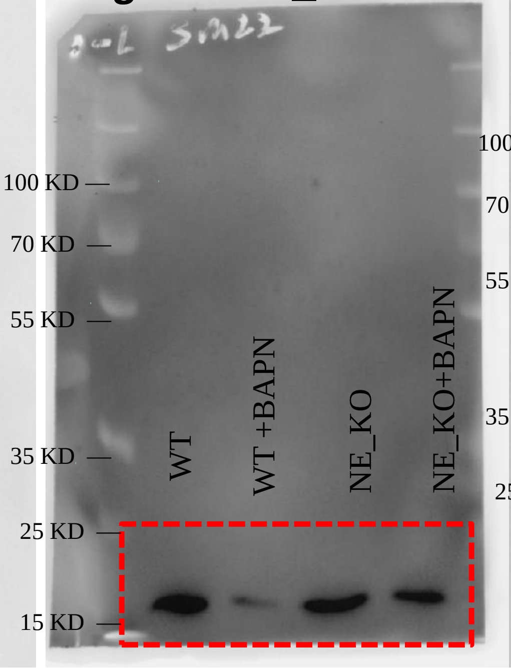

Figure 5E1_tubulin

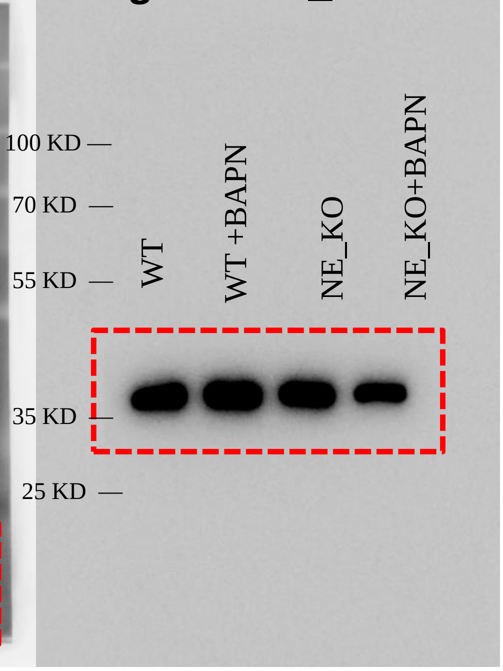

Full unedited blots for Figure 5E1

Figure 5E1_TBL1x

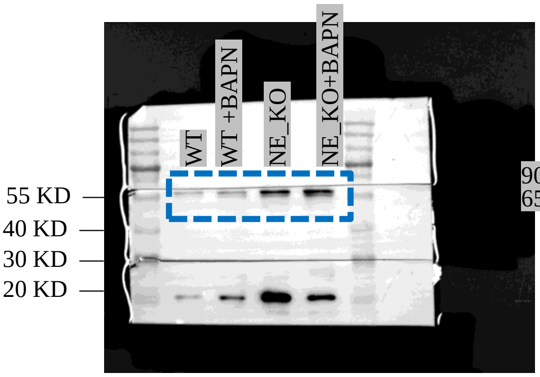

Figure 5E1_MECP2

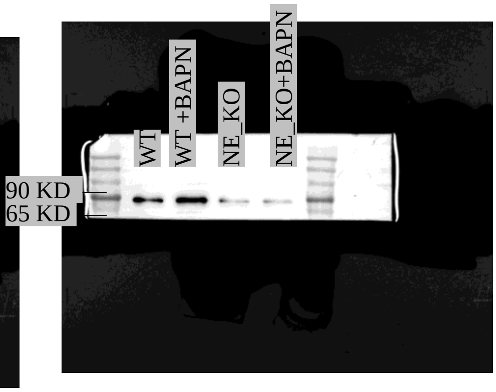

Figure 5E1_SMA

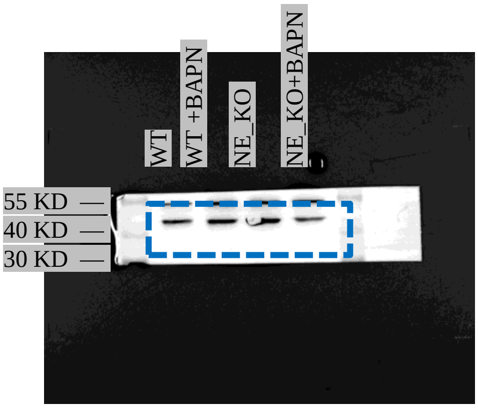

Figure 5E1_SM22

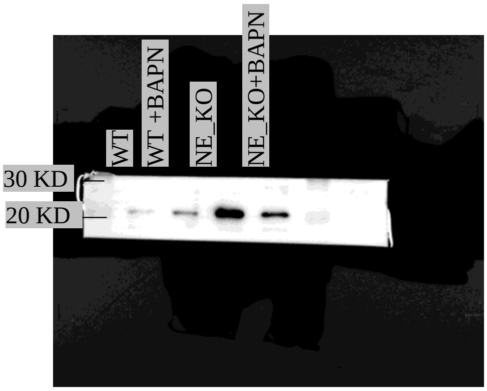

Figure 5E1_tubulin

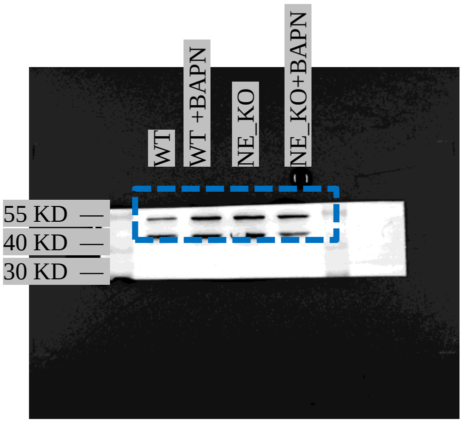

Full unedited blots for Figure 5E1

Figure 5E1_TBL1x

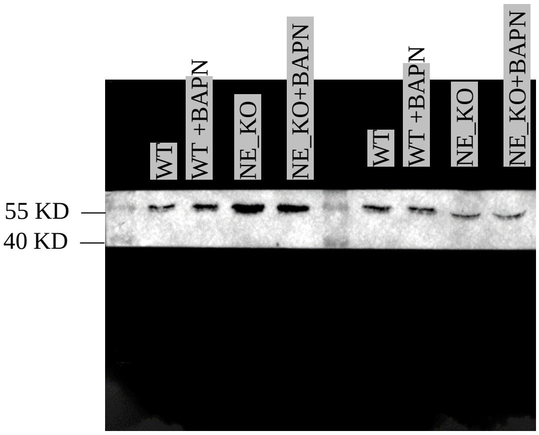

Figure 5E1_MECP2

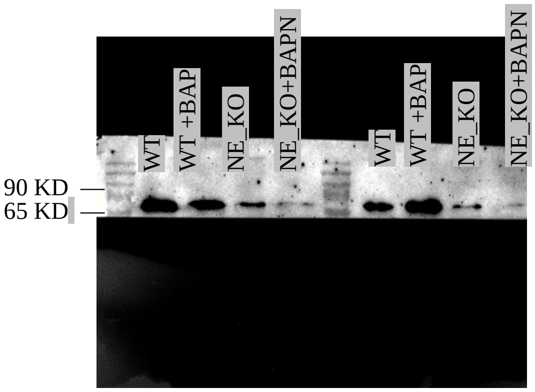

Figure 5E1_SMA

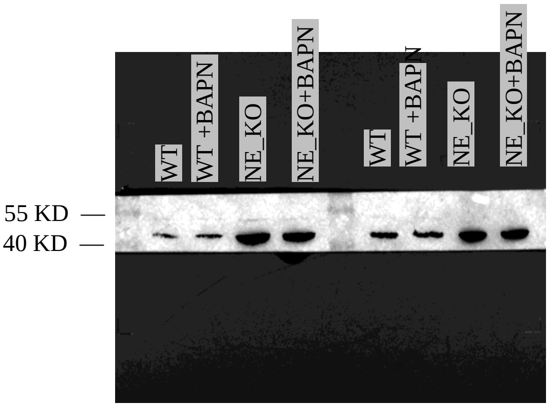

Figure 5E1_SM22

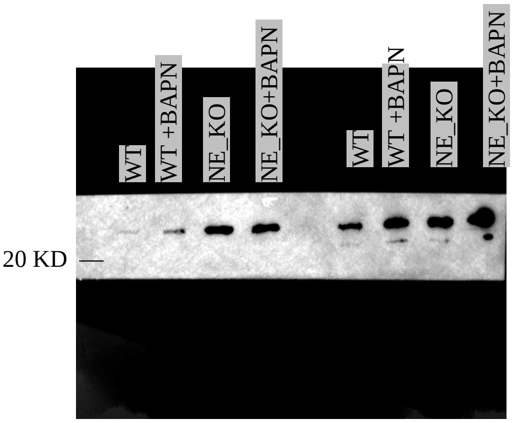

Figure 5E1_tubulin

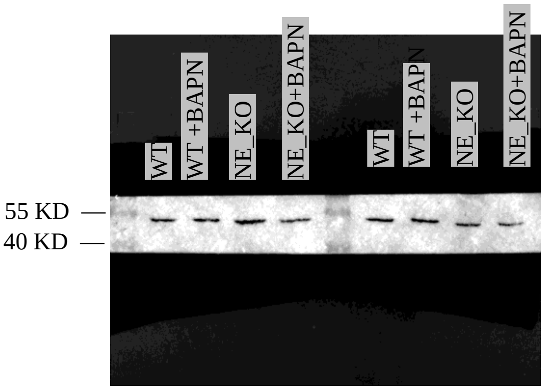

Full unedited blots for Figure 5E1

Figure 5E1_TBL1x

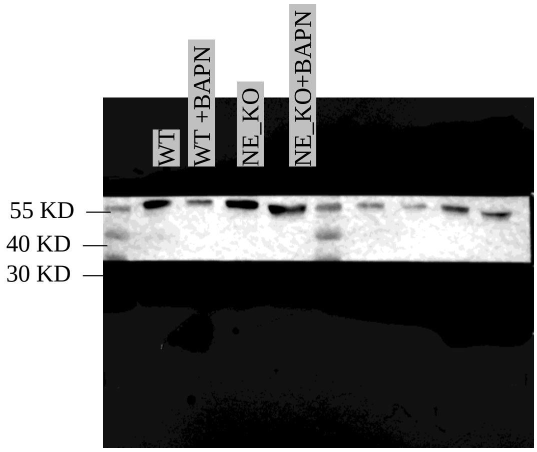

Figure 5E1_MECP2

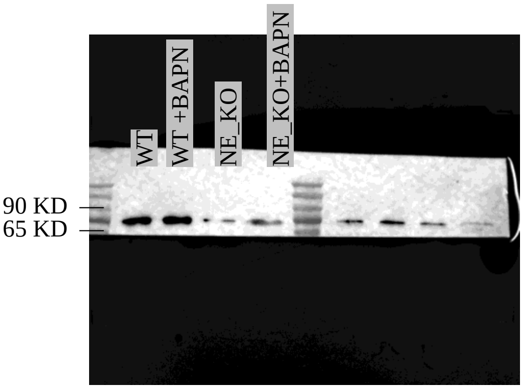

Figure 5E1_SMA

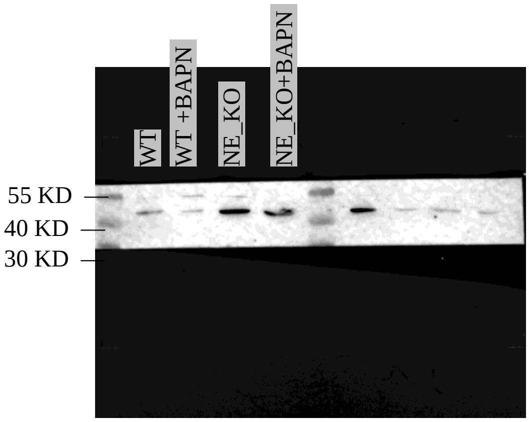

Figure 5E1_SM22

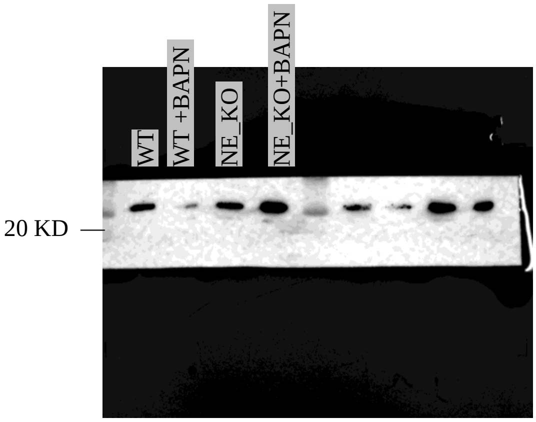

Figure 5E1_tubulin

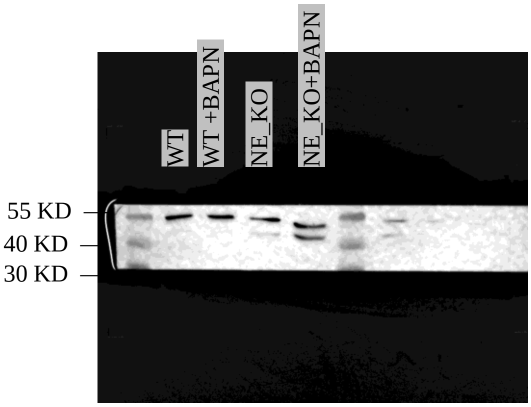

Supplement: Supplementary file 2 [file atv-43-1900-s002.pdf]
